# The oncogenic ADAMTS1–VCAN–EGFR cyclic axis drives anoikis resistance and invasion in renal cell carcinoma

**DOI:** 10.1186/s11658-024-00643-0

**Published:** 2024-09-27

**Authors:** Yu-Ching Wen, Yung-Wei Lin, Kuo-Hao Ho, Yi-Chieh Yang, Feng-Ru Lai, Chih-Ying Chu, Ji-Qing Chen, Wei-Jiunn Lee, Ming-Hsien Chien

**Affiliations:** 1 Department of Urology, Wan Fang Hospital, Taipei Medical University, Taipei, Taiwan; 2 Department of Urology, School of Medicine, College of Medicine and TMU Research Center of Urology and Kidney (TMU-RCUK), Taipei Medical University, Taipei, Taiwan; 3 International Master/PhD Program in Medicine, College of Medicine, Taipei Medical University, Taipei, Taiwan; 4 Graduate Institute of Clinical Medicine, College of Medicine, Taipei Medical University, 250 Wu-Hsing Street, Taipei, 11031 Taiwan; 5 Department of Medical Research, Tungs’ Taichung MetroHarbor Hospital, Taichung, Taiwan; 6 Department of Cancer Biology, Geisel School of Medicine at Dartmouth, Lebanon, NH USA; 7 Department of Medical Education and Research, Wan Fang Hospital, Taipei Medical University, 111 Xinglong Rd., Sec. 3, Wenshan Dist, Taipei, 11696 Taiwan; 8 TMU Research Center of Cancer Translational Medicine, Taipei Medical University, Taipei, Taiwan; 9 Pulmonary Research Center, Wan Fang Hospital, Taipei Medical University, Taipei, Taiwan; 10 Traditional Herbal Medicine Research Center, Taipei Medical University Hospital Taipei, Taipei, Taiwan

**Keywords:** ADAMTS1, VCAN, EGFR, Anoikis, Metastasis, Renal cell carcinoma

## Abstract

**Background:**

Metastasis, the leading cause of renal cell carcinoma (RCC) mortality, involves cancer cells resisting anoikis and invading. Until now, the role of the matrix metalloproteinase (MMP)-related enzyme, A disintegrin and metalloprotease with thrombospondin motifs 1 (ADAMTS1), in RCC anoikis regulation remains unclear.

**Methods:**

The clinical significance of ADAMTS1 and its associated molecules in patients with RCC was investigated using data from the Gene Expression Omnibus (GEO) and TCGA datasets. Human phosphoreceptor tyrosine kinase (RTK) array, luciferase reporter assays, immunoprecipitation (IP) assays, western blotting, and real-time reverse-transcription quantitative polymerase chain reaction (RT–qPCR) were used to elucidate the underlying mechanisms of ADAMTS1. Functional assays, including anoikis resistance assays, invasion assays, and a Zebrafish xenotransplantation model, were conducted to assess the roles of ADAMTS1 in conferring resistance to anoikis in RCC.

**Results:**

This study found elevated ADAMTS1 transcripts in RCC tissues that were correlated with a poor prognosis. ADAMTS1 manipulation significantly affected cell anoikis through the mitochondrial pathway in RCC cells. Human receptor tyrosine kinase (RTK) array screening identified that epidermal growth factor receptor (EGFR) activation was responsible for ADAMTS1-induced anoikis resistance and invasion. Further investigations revealed that enzymatically active ADAMTS1-induced versican V1 (VCAN V1) proteolysis led to EGFR transactivation, which in turn, through positive feedback, regulated ADAMTS1. Additionally, ADAMTS1 can form a complex with p53 to influence EGFR signaling. In vivo, VCAN or EGFR knockdown reversed ADAMTS1-induced prometastatic characteristics of RCC. A clinical analysis revealed a positive correlation between ADAMTS1 and VCAN or the EGFR and patients with RCC with high ADAMTS1 and VCAN expression had the worst prognoses.

**Conclusions:**

Our results collectively uncover a novel cyclic axis involving ADAMTS1–VCAN–EGFR, which significantly contributes to RCC invasion and resistance to anoikis, thus presenting a promising therapeutic target for RCC metastasis.

**Supplementary Information:**

The online version contains supplementary material available at 10.1186/s11658-024-00643-0.

## Background

Renal cell carcinoma (RCC) accounts for approximately 90% of kidney cancer cases in adults, while clear cell RCC (ccRCC) and papillary (p)RCC respectively represent the most common and the second most common histopathological subtypes of RCC [1]. According to GLOBOCAN statistics, RCC has a high incidence and mortality rate with an increasing trend [2]. About 30% of patients have metastatic disease at the time of diagnosis [3]. Although most patients present with localized disease amenable to surgical excision, approximately one-third of patients treated with curative intent will eventually develop metastatic disease recurrence, with a 5-year relative survival rate that ranges about ~0–20% [4]. From the inception of cytokine treatment to the advent of targeted and immune therapies, therapeutic strategies for this lethal stage have evolved in recent years. However, these treatment strategies still fall short, and advanced ccRCC remains a deadly disease [5–7] due to a lack of a clearly defined molecular mechanism of metastasis. Therefore, discovering novel molecular insights, biomarkers, and related treatment targets would have significant clinical impacts on managing patients with ccRCC.

Anoikis is a type of programmed cell death that is initiated immediately after cell-extracellular matrix (ECM) interactions are disrupted, when cells detach from the original ECM [8]. Anoikis is regulated by two pathways termed the intrinsic and extrinsic pathways. The intrinsic pathway is triggered by translocation of the proapoptotic Bax and Bak proteins to the outer mitochondrial membrane, which increases the mitochondrial permeability and leads to the formation of the apoptosome. Death receptors, such as those in the tumor necrosis factor receptor superfamily, initiate the extrinsic pathway by binding to ligands, which in turn leads to the formation of a death-inducing signaling complex [9, 10]. Both pathways terminally converge to activation of caspases and downstream molecular pathways to execute anoikis. Development of anoikis resistance is a critical mechanism in cancer and contributes to distal tumor metastasis [11]. For example, gastric, colon, and lung cancers were reported to induce anoikis resistance and metastasis through epidermal growth factor receptor (EGFR) signaling [12–14]. Melanoma cells were reported to promote anoikis resistance via upregulating N-cadherin [15]. While associations of anoikis resistance with metastasis and its underlying mechanisms have been documented in various cancer types, such reports are rare in RCC.

ECM enzymes such as matrix metalloproteinases (MMPs) were reported to be correlated with anoikis resistance in different cancer types. For example, MMP7 was shown to play an essential role in anoikis resistance of chondrosarcoma cells [16]. Upregulation of MMP-2 and MMP-9 was reported to be involved in sine oculis homeobox homolog 1-induced anoikis resistance and invasion in cervical cancer cells [17]. Likewise, MMP-11 was shown to increase anoikis resistance in lobular carcinoma breast cells [18]. MMPs from the ADAMTS (A disintegrin and metalloprotease with thrombospondin motifs) family were also implicated in ECM remodeling events that occur during cancer development and progression [17]. Among these, ADAMTS1 is the most extensively studied member that regulates cancer progression [19] via targeting specific substrates within the ECM, such as proteoglycans [20] and growth factors [21]. So far, the functions of ADAMTS1 in cancer progression remain diverse across various cancer types [22]. While previous research indicated an increase in ADAMTS1 expression in kidney cancer [19], there is limited information regarding its potential clinical predictive significance in RCC, particularly with regards to the influence of ADAMTS1 on RCC’s resistance to anoikis and the underlying mechanisms of this phenomenon.

Herein, we discovered that ADAMTS1 expression was significantly upregulated in ccRCC tissues and was correlated with poor prognoses. ADAMTS1 overexpression has the potential to enhance anoikis resistance of and invasion by ccRCC cells in a protease enzyme activity-dependent manner. Additionally, the impact of ADAMTS1 on these biological behaviors in ccRCC cells relies on the proteolysis of the ECM proteoglycan, versican (VCAN), by ADAMTS1, resulting in induction of EGFR signaling. Activated EGFR signaling, in turn, exhibits positive feedback regulation on ADAMTS1. Consequently, the cyclic increase in ADAMTS−VCAN−EGFR axis-induced cell anoikis resistance and invasion may represent a novel therapeutic target for ccRCC.

## Materials and methods

### Materials

Recombinant human ADAMTS1 (2197-AD) containing a zinc-dependent metalloprotease (ZnMc) domain was purchased from R&D Systems (Minneapolis, MN). Recombinant human ADAMTS1 (RPB973Hu02) containing thrombospondin (TSP) type I domains was purchased from Cloud-Clone (Houston, TX). Pifithrin-α (PFT-α, P4359), dimethyl sulfoxide (DMSO, D2650), and crystal violet (C0775) were purchased from Sigma-Aldrich (St. Louis, MO). Antibodies employed in the western blot, dot blot, and coimmunoprecipitation (Co-IP) analyses were as follows: Bid (no. 2002), Bad (no. 9239), Bak (no. 12,105), Bim (no. 2933), poly(ADP ribose polymerase (PARP; no. 9532), cleaved PARP (no. 5625), phosphorylated (p)-EGFR (no. 3777), p-Src (no. 2101), p-Akt (no. 9271), p-extracellular signal-regulated kinase (ERK; no. 4370), p-signal transduction and activator of transcription 3 (Stat3; no. 9145), Akt (no. 9272), ERK (no. 4695), Stat3 (no. 9139; Cell Signaling Technology, Danvers, MA); EGFR (sc-03), Wilm’s tumor 1 (WT1; sc-7385), Lamin A/C (sc-7292), p-ErbB2 (SC-81507), ErbB (SC-33684), p53 (sc-126; Santa Cruz Biotechnology, Santa Cruz, CA); versican V1 (DPEAAE) (ab19345), Bcl-2 (ab32124; Abcam, Cambridge, MA); GAPDH (60,004–1-Ig), α-tubulin (66,031–1-Ig; Proteintech, Chicago, IL); ADAMTS1 (AF5867; R&D Systems); and c-Jun (GTX101135; GeneTex, Irvine, CA).

### Data collection from bioinformatics analyses

Messenger RNA (mRNA) expression levels and corresponding survival data of patients from the Cancer Genome Atlas Kidney Renal Clear Cell Carcinoma (TCGA-KIRC) were obtained from UCSC Xena (https://xena.ucsc.edu/). The cDNA microarray data, GSE53757, containing 72 paired RCC tumors and normal renal tissues, and the single cell (SC) RNA scequecing data of GSE159115, comprising eight renal tumor specimens and six benign human kidney specimens from RCC were acquired from Gene Expression Omnibus (GEO) datasets. The probe used for analyzing ADAMTS1 was 222162_s_at. Violin plots for detecting ADAMTS1, EGFR, and tissue inhibitor of metalloproteinase 3 (TIMP3) expressions in kidney cancer tissues were retrieved from available online data at TNMplot (https://tnmplot.com/analysis/)(23). For TCGA RNA sequencing data, gene expressions were normalized by fragments per kilobase per million (FPKM) and log_2_-transformed. To analyze the correlation between anoikis resistance and the expression of ADAMTS10, EGFR, and VCAN, we utilized the “negative regulation of anoikis” pathway derived from the gene ontology database. We performed a single sample gene set enrichment analysis (ssGSEA) to calculate the score of this pathway for each patient in the TCGA-KIRC dataset. A Spearman correlation analysis was then conducted to evaluate the relationship between the “negative regulation of anoikis” pathway and the expression levels of ADAMTS10, EGFR, and VCAN. For the single-cell RNA sequencing (scRNA-seq) analysis, a differentially expressed gene analysis was performed to compared the expression of ADAMTS1, EGFR, and VCAN between tumor and benign epthelial cells. We utilized a uniform manifold approximation and projection (UMAP) plot to demonstrate the distribution of tumor and benign cells based on their transcription profiles from the scRNA-seq data.

### Cell lines and cell culture

The Caki-1, 786-O, and Achn RCC cell lines were purchased from American Type Culture Collection (ATCC, Manassas, VA). Cells were maintained in minimum essential medium (MEM) (Caki-1 and Achn) or RPMI-1640 medium (786-O) with 10% fetal bovine serum, 2 mM l-glutamine, 100 U/mL penicillin, and 100 μg/mL streptomycin at 37 °C in 5% CO_2_.

### Construction of the E402Q mutation of ADAMTS1

The pLex-MCS-ADAMTS1 expression construct was kindly provided by Dr. Tsang-Chih Kuo (National Taiwan University, Taipei, Taiwan). Metalloproteinase activity in ADAMTS1 was inactivated by generating a mutation of glutamate (Glu, E) into glutamine (Gln, Q) on amino acid 402 using a QuikChange Lightning Site-Directed Mutagenesis Kit (Agilent Technologies, Santa Clara, CA). The following primers were used to generate E402Q: ADAMTS1-E402Q-F: GTT AAA CAC GTG GCC TAA CTG ATG GGC TGT GGT GAA GGC and ADAMTS1-E402Q-R: GCC TTC ACC ACA GCC CAT CAG TTA GGC CAC GTG TTT AAC.

### Establishment of gene knockdown (KD) and overexpression of RCC cells

Short hairpin (sh) RNAs for ADAMTS1, VCAN, and EGFR were obtained from the the RNA Technology Platform and Gene Manipulation Core Facility at Academic Sinica (Taipei, Taiwan), including targeting sequences for shADAMTS1 (CCA CAG GAA CTG GAA GCA TAA), shVCAN (ATG GAT GTG TTC AAC CTT AAT), and shEGFR (GCC AAG CCA AAT GGC ATC TTT). To produce the lentivirus, 293 T packaging cells were cotransfected with shRNA constructs or pLex-MCS-ADAMTS1 together with pCMVDR8.91 and pMD.G. according to protocols from our previous study [24]. After overnight incubation, the transfection medium was removed and replaced with fresh culture medium. Viral supernatants were collected at 24 and 48 h. Then, RCC cells were infected with fresh lentivirus-containing medium (supplemented with 8 μg/mL polybrene) for 24 h and subjected to the following assays.

The pcDNA3-TIMP3 plasmid was acquired from Dr. Shun-Fa Yang (Chung Shan Medical University, Taichung, Taiwan). RCC cells were transfected with either 2 μg of an empty vector or pcDNA3-TIMP3 using the Lipofectamine 3000 Transfection Reagent (Invitrogen, Carlsbad, CA) for 6 h, following the manufacturer’s guidelines.

### Western blot analysis

Cell lysates of the experimental groups were extracted using PRO-PREPTM protein extraction buffer (iNtRON Biotechnology, Seongnam, Korea) supplemented with protease inhibitor cocktails. Proteins were quantified using bovine serum albumin (BSA) and Coomassie Brilliant Blue G-250 dye (Bio-Rad protein assay). Approximately ~15–30 µg of protein was then loaded and separated via sodium dodecylsulfate polyacrylamide gel electrophoresis (SDS–PAGE), followed by transfer onto polyvinylidene difluoride (PVDF) membranes. Specific primary antibodies were used to detect targeted proteins. After three washes of blots in Tris Buffered Saline with Tween 20 (TBST), incubation with a secondary antibody at room temperature was performed. Finally, chemiluminescence was utilized for detection.

### Anoikis resistance assay

Caki-1 and 786-O cells, modified for ADAMTS1, EGFR, or VCAN expression, or exposed to ADAMTS1-indicative recombinant proteins, were initially plated at a density of 5 × 10^4^ cells/well in six-well ultralow-attachment plates for either 24 or 48 h. Afterwards, suspended cells were harvested and reseeded into three separate wells of 96-well plates for an additional ~3– 4 h. Subsequently, cell viability was evaluated using the CCK-8 viability assay (Abcam).

### Zebrafish xenotransplantation model

Transgenic Tg (fli1: EGFP) zebrafish were utilized to establish a zebrafish xenograft model to assess the RCC distant metastatic potential. A total of 400 Caki-1 or 786-O RCC cells expressing a vector control, ADAMTS1-overexpression, ADAMTS1 + shVCAN, or ADAMTS1 + shEGFR, labeled with CM-DiI dye (ThermoFisher Scientific, Waltham, MA), were injected into a zebrafish yolk sac under anesthesia with 0.1 mg/mL tricaine. Zebrafish injected with tumor cells were maintained at 34 °C for a specified duration. Fluorescence signals present in the trunk and end-tail of the fish, indicating RCC cancer cell metastatic activity, were monitored daily using a Zeiss Axiophot fluorescence microscope (Carl Zeiss Microimaging, Gottingen, Germany). Distant metastasis signals were quantified using ImageJ software (National Institutes of Health, Bethesda, MD).

### Human phosphoreceptor tyrosine kinase (RTK) array

The phosphorylation status of RTKs was analyzed by collecting 300 μg of total protein lysates from ADAMTS1-manipulated Caki-1 cells. Subsequently, proteins were harvested and subjected to incubation with a membrane-based RTK antibody array (ARY001B; R&D) according to the manufacturer’s instructions. Following the reaction with specific antibodies and target proteins, signals were detected using a chemiluminescent horseradish peroxidase (HRP) substrate. Spot densities were then normalized against respective reference array spots and further against the controls.

### Real-time reverse-transcription quantitative polymerase chain reaction (RT–qPCR)

mRNA was isolated from specified experimental groups of RCC cells using Trizol (Invitrogen, Carlsbad, CA). Approximately 1 μg of total RNA was then employed for reverse transcription to complementary DNA (cDNA) using an iScript™ cDNA Synthesis Kit (Bio-Rad, Hercules, CA). The resulting cDNA was subsequently utilized in RT–qPCR assays with a TOOLS 2 × SYBR qPCR Mix kit (BIOTOOLS, New Taipei City, Taiwan), as detailed in our prior study[25]. Primers used in the RT–qPCR are listed as follows: ADAMTS1_F: TTT TGT TCA CAC ACT TGC CGT T and ADAMTS1_R: CAG TGC CAG TTT ACA TTT GGG G; VCAN_F: AAC GGC TTT GAC CAG TGC GA and VCAN_R: ATC AGG GGG AGG GAA GCC TG; TIMP3_F: ACC GAG GCT TCA CCA AGA TG and TIMP3_R: CCC CGT GTA CAT CTT GCC AT; EGFR_F: AGG CAC GAG TAA CAA GCT CAC and EGFR_R: ATG AGG ACA TAA CCA GCC ACC; and GAPDH_F: 5′-CTGGAGAAACCTGCCAAGTATGAT-3′ and GAPDH_R: 5′-TTCTTACTCCTTGGAGGCCATGTA-3′. We also designed the following primer set to detect expression levels of VCAN isoforms, V0_F: CAG CCC CCA GCA AGC ACA AAA TT and V0_R: TCA GCC ATT AGA TCA TGC ACT GG; V1_F: GAA CCC TGT ATC GTT TTG AGA ACC and V1_R: TCA GCC ATT AGA TCA TGC ACT GG; V2_F: CAG CCC CCA GCA AGC ACA AAA TT and V2_R: GCA TAC GTA GGA AGT TTC AGT AGG; and V3_F: GAA CCC TGT ATC GTT TTG AGA ACC and V3_R: GCA TAC GTA GGA AGT TTC AGT AGG.

### Transwell invasion assay

An invasion assay was conducted using 24-well Transwell assays (with an 8-μm pore size; Corning Costar, Corning, NY). Approximately 5 × 10^4^ RCC cells were seeded into the upper chamber coated with Matrigel (BD Biosciences, Bedford, MA) and incubated for 48 h. After incubation, cells were fixed with methanol for 20 min and stained with 0.5% crystal violet for an additional ~3–4 h. Cells in the upper chamber were carefully removed using cotton buds. Quantification was performed by counting invaded cells in five individual microscopic fields (×100 magnification) of stained cells.

### Dot blot assay

Conditioned medium (CM) from specified RCC cells was collected and centrifuged at a minimum speed of 13,000 rpm to remove cell debris. Around 300 μL of the supernatant was carefully loaded onto nitrocellulose membranes using a 96-well GFE960 Dot Blotter (GenePure, Taichung, Taiwan) and drawn slowly through the membrane by a suction pump. Antibody hybridization and signal detection were carried out following the western blot protocol.

### Luciferase reporter assay

An EGFR promoter construct was purchased from GeneCopoeia (clone HPRM45993) (Rockville, MD) and transfected into RCC cells with 1 μg plasmid DNA for the final 48 h to examine promoter activity, which stably manipulated ADAMTS1 expression. For CM treatment, RCC cells were first transfected with reporter constructs for 24 h, and the cells were then further treated with the indicated CM for another 24 h. Medium from RCC cells was collected, and Gaussia luciferase activity was determined using a Secrete-Pair™ Gaussia Luciferase Assay kit (GeneCopoeia). In the meantime, cell lysates were harvested to determine Renilla luciferase activity as the internal control by a luciferase assay kit (Promega, Madison, WI).

### Subcellular fractionation

Isolation of cytoplasmic fractionation was achieved using a cytosolic lysis buffer (10 mM HEPES, 1.5 mM MgCl_2_, 10 mM KCl, 1 mM DTT, and 1% NP-40), followed by centrifugation at 13,000 rpm for 5 min. The resulting supernatant, containing cytosolic proteins, was collected. The pellet was then lysed using PRO-PREPTM protein extraction buffer and subjected to an additional centrifugation step at 13,000 rpm for 15 min. Each fraction obtained was separated by SDS–PAGE and probed for ADAMTS1. Fraction purity was evaluated by detecting GAPDH for the cytoplasmic fraction and lamin A/C for the nuclear fraction.

### Immunoprecipitation (IP) assay

Total protein from RCC cells was extracted using NETN buffer (20 mM Tris at pH 8.0, 100 mM NaCl, 1 mM EDTA, and 0.5% NP-40). Approximately 1.5 mg of protein was incubated with an anti-ADAMTS1 antibody (AF5867) at 4 °C for 24 h, followed by incubation with Protein A Sepharose beads for ~1–2 h. After washing the beads five times with NETN buffer, bound proteins were eluted and boiled with 6× sample buffer. The resulting bound proteins were then analyzed by western blotting.

### Statistical analysis

Values from both in vitro and in vivo studies are presented as the mean ± standard deviation (SD). A Student’s *t*-test was employed for data analysis when comparing two groups. In the analysis of clinical data, Spearman correlations were used to assess the correlation between ADAMTS1 and EGFR or VCAN expression. Associations of gene expressions with overall survival (OS) and disease-specific survival (DSS) were evaluated using a log-rank test. For comparisons involving three groups, a one-way analysis of variance (ANOVA) followed by Tukey’s post hoc test was conducted to compare each group. The expression cutoff to define high or low expression groups was determined based on the minimum log-rank test *p* value. Gene expressions from paired tumor/normal tissues were compared using a paired *t*-test.

## Results

### Expression levels of ADAMTS1 and its prognostic potential in RCC

**Fig. 1:**
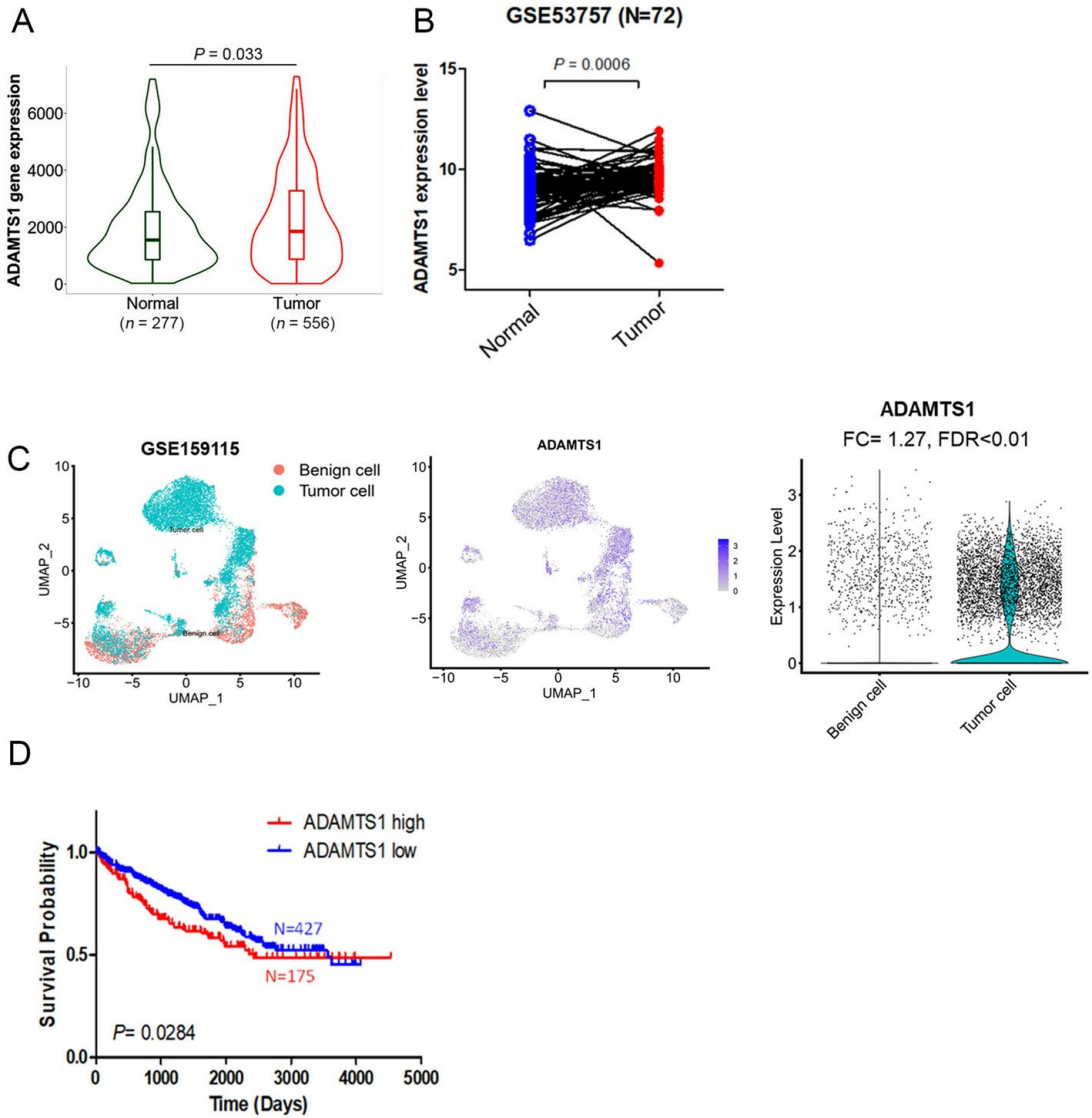
Elevated ADAMTS1 expression in renal cell carcinoma (RCC) tissues is associated with a poor prognosis. **A** ADAMTS1 mRNA levels in RCC tissues were significantly higher than those in normal renal tissues (*p* = 0.033) as determined by TNMplot (https://tnmplot.com/analysis/). **B** ADAMTS1 expression was assessed in 72 matched clear cell (cc)RCC tissues and their corresponding normal tissues using data from the GSE53757 ccRCC expression microarray dataset. **C** The left panel of the Uniform Manifold Approximation and Projection (UMAP) plot illustrates the distribution of tumor cells and benign epithelial cells based on single-cell RNA sequencing (scRNA-seq) profiles. The UMAP plot in the middle panel shows the expression distribution of ADAMTS1. The violin plot in right panel indicates that ADAMTS1 exhibits a significant elevation in tumor cells compared with benign epithelial cells, with a fold change (FC) of 1.27 and a false discovery rate (FDR) of less than 0.01. **D** Kaplan–Meier analysis depicts overall survival (OS) rates in patients with ccRCC based on high or low ADAMTS1 expression using data from TCGA

To elucidate the clinical relevance of ADAMTS1 in patients with RCC, we initially analyzed its expression levels in 277 noncancerous tissues and 566 RCC samples obtained from gene chip data provided by the TNMplot database. Significantly higher ADAMTS1 expression was observed in tumors compared with normal tissues (Fig. 1A). Additionally, in the ccRCC cohort obtained from the GSE53757 dataset of the GEO database, we also found significantly higher ADAMTS1 levels in tumors than in adjacent normal tissues (Fig. 1B). In the scRNA sequencing dataset GSE159115, ccRCC tumor cells also exhibited heightened expression compared with benign epithelial cells (Fig. 1C). Furthermore, the Kaplan–Meier plot revealed that patients with ccRCC with ADAMTS1^high^ levels in tumors had a poorer prognosis (OS periods) than patients with ADAMTS1^low^ (Fig. 1D). These clinical data imply the oncogenic roles of ADAMTS1 in RCC development.

### ADAMTS1 expression promotes anoikis resistance in RCC cells

**Fig. 2:**
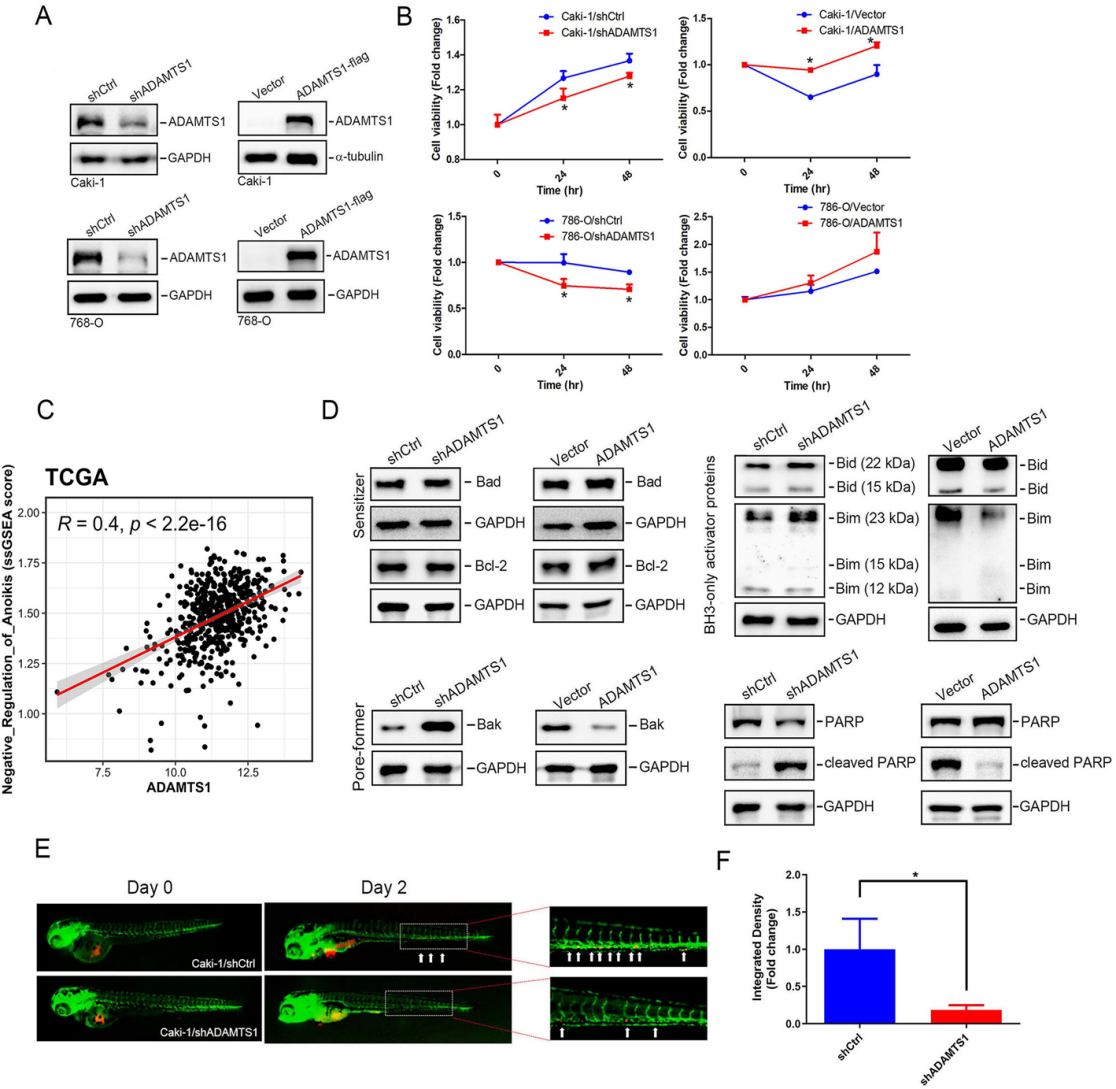
ADAMTS1 expression promotes anoikis resistance of renal cell carcinoma (RCC) via inhibiting Bid, Bim, and Bak. **A** A western blot analysis was conducted to assess ADAMTS1 expression in Caki-1 and 786-O cells following transduction with either ADAMTS1 short hairpin (sh)RNA (left) or an ADAMTS1-expressing vector (right). **B** Cell viability of suspended Caki-1 and 786-O cells was evaluated using a CCK8 assay at 24 and 48 h post-stable overexpression (right) or knockdown (left) of ADAMTS1. Data are presented as the mean ± SD of three independent experiments. * *p* < 0.05, compared with the control group. **C** Dot plot demonstrated the correlation between the single sample gene set enrichment analysis (ssGSEA) score of “negative regulation of anoikis” and ADAMTS1 expression in TCGA-KIRC patients. A Pearson correlation was performed to evaluate their association and significance. **D** Western blot analysis of intrinsic apoptosis-related proteins (Bad, Bcl-2, Bak, Bid, Bim, and PARP) in suspended Caki-1 cells manipulated with ADAMTS1. GAPDH served as a loading control. **E** and **F** Dissemination of RCC cells in zebrafish embryos. Caki-1 cells with ADAMTS1-knockdown were implanted into zebrafish embryos at 48 h post fertilization. Tumor cell dissemination was observed at 2 days post injection (dpi), with disseminated tumor foci indicated by white arrowheads on the trunk and end-tail (**E**). Integrated densities of Caki-1 metastatic tumor cells in the zebrafish trunk and end-tail at 2 dpi were quantified, with the mean value of the integrated density in the shCtl group set to onefold (**F**)

Our previous studies demonstrated that RCC invasion is significantly facilitated by ADAMTS1 [26], highlighting the potential role of ADAMTS1 in driving anoikis resistance, a critical factor for tumor cell invasion and metastasis [11]. To investigate this further, we employed lentiviral-based RNA KD and overexpression approaches to establish stable KD and overexpression of ADAMTS1 in Caki-1 (Fig. 2A, top) and 786 cells (Fig. 2A, bottom). ADAMTS1-manipulated cells were cultured in suspension for ~24–48 h to allow spheroid formation. Subsequently, cells were plated under adherent conditions to assess cell viability, which served as a surrogate for anoikis resistance. As depicted in Fig. 2B, we observed that forced and decreased ADAMTS1 expression respectively promoted and suppressed the cell viability of RCC cells cultured in suspension for 24 or 48 h. Furthermore, ADAMTS1 is positively associated with the ssGSEA score of “negative regulation of anoikis” pathway in TCGA-KIRC patients (Fig. 2C), suggesting that ADAMTS1 is crucial for anoikis resistance in RCC. To delve into the molecular mechanism of ADAMTS1-mediated anoikis resistance, we analyzed expressions of the antiapoptotic Bcl-2 protein and proapoptotic family members involved in mitochondrial apoptosis, including sensitizer (Bad) and activator (Bid and Bim) BH3 proteins, and the Bak pore-forming effector. We observed that ADAMTS1 overexpression in Caki-1 cells decreased Bim, Bid, Bak, and PARP cleavage under suspension conditions. However, there was no change in Bcl-2 or Bad expression (Fig. 2D). In contrast, ADAMTS1-KD showed the opposite outcome in Caki-1 (Fig. 2D) and 786-O cells (Additional file 1: Supplementary Fig. 1). These results suggest that ADAMTS1 modulates cell anoikis through the mitochondrial pathway in RCC cells. We proceeded to investigate the in vivo effects of ADAMTS1 on RCC metastasis. Herein, we established a zebrafish xenograft metastasis model by transplanting DiI dye-labeled Caki-1 cells expressing shADAMTS1 or shCtrl into transgenic Tg (fli1: EGFP) zebrafish embryos, resulting in extensive dissemination of tumor cells. At 2 days post injection, metastatic dissemination of inoculated cells was captured using fluorescence microscopy (Fig. 2E). In fish that were inoculated with ADAMTS1-KD Caki-1 cells, trunk and end-tail dissemination of cells had dramatically decreased compared with fish that were inoculated with control cells (Fig. 2F), suggesting ADAMTS1 can promote RCC metastasis in vivo.

### EGFR transactivation plays a critical role in ADAMTS1-modulated anoikis resistance and invasive ability of RCC cells

**Fig. 3:**
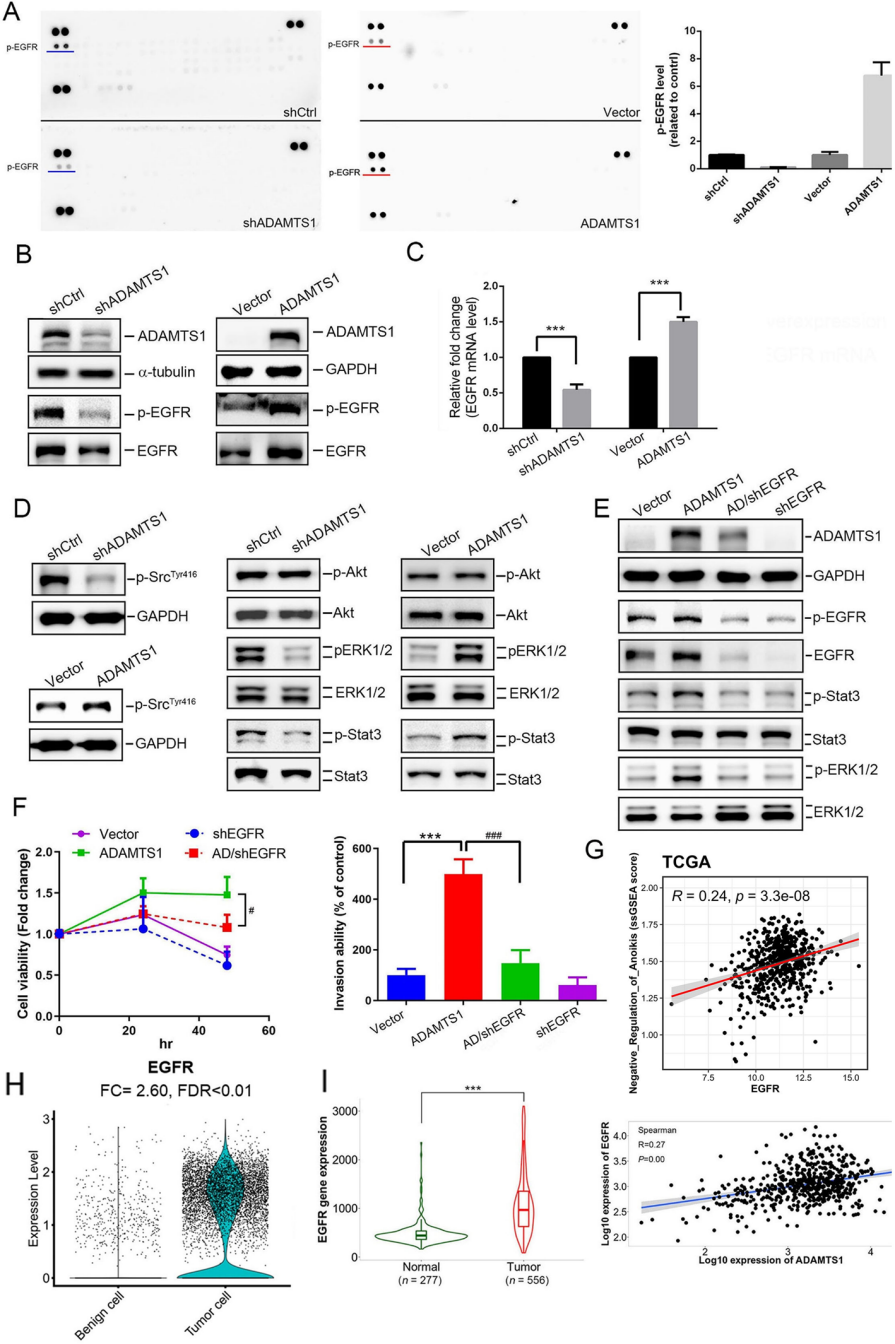
ADAMTS1 expression activates epidermal growth factor receptor (EGFR) signaling cascades to promote anoikis resistance of and invasion by Caki-1 renal cell carcinoma (RCC) cells. **A** Differential expression levels of phosphorylated receptor tyrosine kinases (RTKs) in cell lysates from AMAMTS1-manipulated Caki-1. An antibody array (R&D Systems) was used to detect 49 different phosphorylated human RTKs. The left panel shows representative array blots at a short exposure time (30 s). The right panel shows a quantitative analysis of phosphorylated (p)-EGFR using a densitometer. Values are presented as the mean ± SD. *n* = 2. **B**–**D** Caki-1 cells were subjected to knockdown or overexpression of ADAMTS1 to assess protein levels of p-EGFR and EGFR (**B**), as well as their downstream signaling cascades (**D**), using western blotting. Additionally, mRNA levels of the EGFR were analyzed using a real-time qPCR (**C**). (**E** and **F**) EGFR shRNA was transfected into ADAMTS1-overexpressing Caki-1 cells as indicated, and cell lysates were collected to detect expressions or phosphorylation of ADAMTS1, EGFR, ERK, and Stat3 (**E**). Additionally, cell viability in suspended conditions (**F**, left) and the cell invasive ability (**F**, right) were respectively evaluated by CCK8 and Matrigel invasion assays. Multiples of differences are presented as the mean ± SD of three independent experiments. ***p < 0.001, compared with the control group; ^#^*p* < 0.05, ^###^*p* < 0.001, compared with the ADAMTS1-overexpressing only group. (**G**) Dot plot demonstrated the correlation between the single sample gene set enrichment analysis (ssGSEA) score of “negative regulation of anoikis” and EGFR expression in TCGA-KIRC patients. A Pearson correlation was performed to evaluate their association and significance. (**H**) The violin plot indicates that EGFR expression is significantly higher in tumor cells compared with benign epithelial cells, with a fold change (FC) of 2.60 and a false discovery rate (FDR) of less than 0.01. (**I**) Left: EGFR mRNA levels in RCC tissues were significantly higher than those in normal renal tissues (*p* < 0.001). Right: a positive correlation between ADAMTS1 and EGFR expression in RCC tissues. The kidney cancer dataset was retrieved from the TNMplot database.

Activation of growth factor receptors, such as EGFR, TrkB receptor, insulin receptor, platelet-derived growth factor receptor (PDGFR), vascular endothelial growth factor receptor (VEGFR), and Met/hepatocyte growth factor receptor (HGFR), was reported to suppress anoikis [8, 11]. To further investigate the possible mechanisms participating in ADAMTS1-mediated anoikis resistance and invasive ability of RCC cells, we dissected the effect of ADAMTS1 on growth factor receptors using human p-RTK arrays (Fig. 3A, left). Among the 49 phosphorylated RTKs spotted in duplicate, it was shown that EGFR was the most downregulated with a short exposure time in ADAMTS1-depleted Caki-1 cells compared with control cells. In contrast, p-EGFR was dominantly upregulated in ADAMTS1-overexpressing Caki-1 cells compared with control cells (Fig. 3A, right). A western blot analysis was performed to further validate whether ADAMTS1 can modulate EGFR activation in RCC cells. Herein, we observed that p-EGFR (Tyr 1068) was respectively decreased and increased after ADAMTS1 KD and overexpression in Caki-1, 786-O, or ACHN cells (Fig. 3B, Additional file 1: Supplementary Fig. 2A, B). Moreover, downstream signals of activated EGFR, including Src, ERK, and Stat3, were also respectively decreased and increased after ADAMTS1 KD and overexpression in Caki-1 and 786-O cells (Fig. 3D, Additional file 1: Supplementary Fig. 2D). To our surprise, manipulation of ADAMTS1 in RCC cells not only influenced the phosphorylation but also affected the mRNA and protein expression levels of the EGFR (Fig. 3B, C, Additional file 1: Supplementary Fig. 2A–C). Next, to dissect the role of the EGFR in ADAMTS1-modulated RCC progression, we knocked down the EGFR using an EGFR-specific shRNA in Caki-1/ADAMTS1 cells. We observed that EGFR-KD dominantly reversed ADAMTS1 overexpression-induced upregulation of p-EGFR and EGFR (Fig. 3E), as well as increases in anoikis resistance (Fig. 3F, left) and the invasive ability (Fig. 3F, right). Similar phenomena were also observed in 786-O cells (Additional file 1: Supplementary Fig. 2E–G). On the other hand, inhibiting the EGFR signaling cascades by treating EGFR inhibitor, cetuximab, also abolished the ADAMTS1-induced anoikis resistance (Additional file 1: Supplementary Fig. 3). Additionally, EGFR expression is positively correlated with the ssGSEA score of “negative regulation of anoikis” pathway in TCGA-KIRC patients, similar to ADAMTS1 (Fig. 3G). These results suggest dependence on the EGFR for ADAMTS1-regulated cell-anoikis resistance and invasive ability. Moreover, EGFR-KD also reversed upregulation of ADAMTS1 in ADAMTS1-overexpressing Caki-1 and 786-O cells (Fig. 3E, Additional file 1: Supplementary Fig. 2E), suggesting that the EGFR might exert feedback regulation on ADAMTS1 expression in RCC cells. In the scRNA sequencing data, GSE159115, EGFR is likewise upregulated in tumor cells compared with benign epithelial cells (Fig. 3H). From the same TNMplot database described above, we observed significantly higher EGFR levels in RCC tissues compared with normal tissues (Fig. 3I, left), and EGFR expression in RCC was significantly correlated with ADAMTS1 expression (Fig. 3I, right).

## Secreted versican is essential for cyclic ADAMTS1–EGFR axis-promoted anoikis resistance and invasion of RCC cells

**Fig. 4:**
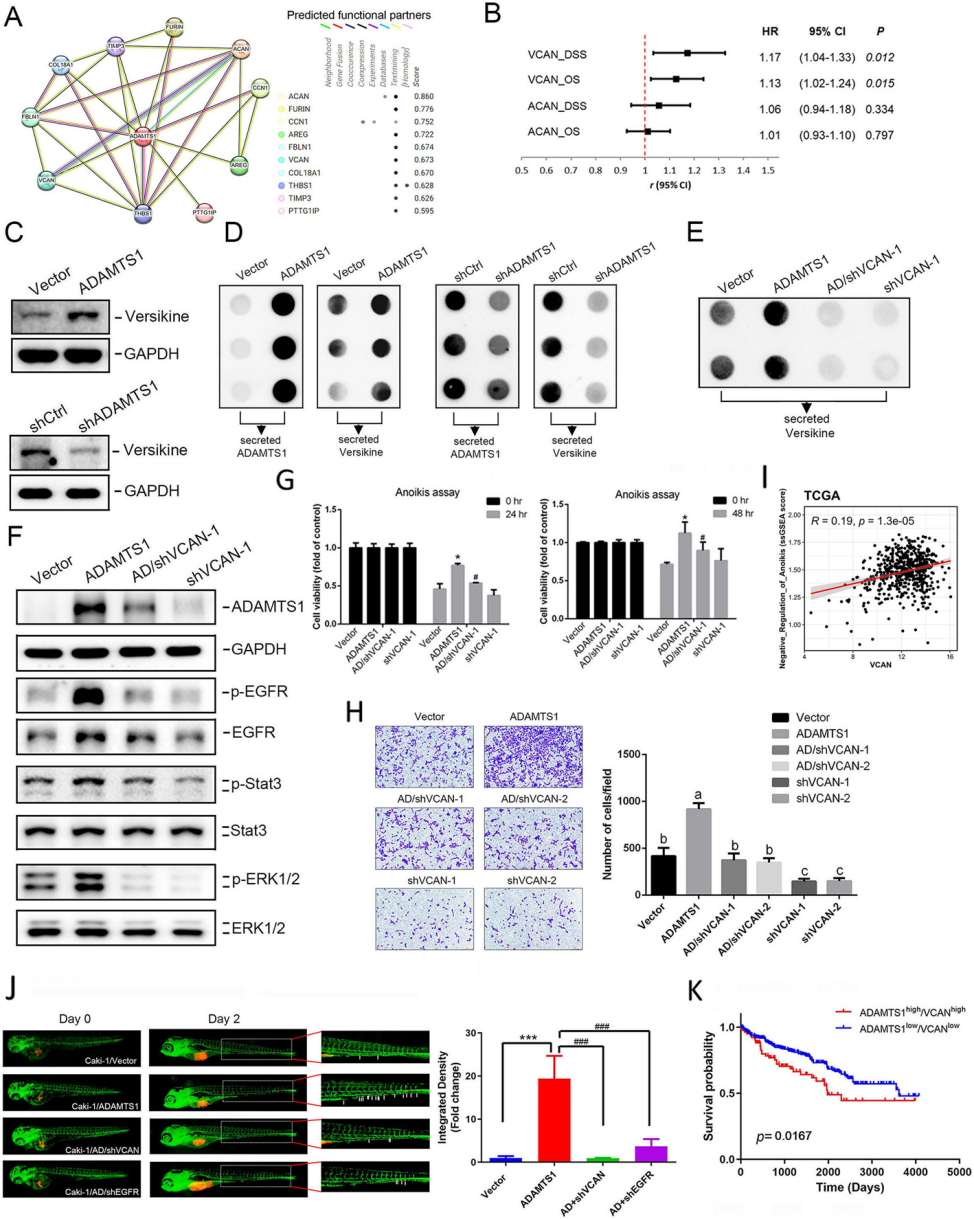
Versican (VCAN) cleavage is critical for ADAMTS1-mediated epidermal growth factor receptor (EGFR) activation, anoikis resistance, invasion, and metastasis of renal cell carcinoma (RCC) cells. **A** ADAMTS1 protein–protein interaction network of ten differentially expressed genes from the STRING database. **B** The forest plot illustrates hazard ratios (HRs) and 95% confidence intervals, examining the correlation between candidate genes [*ACAN* (aggrecan) and *VCAN*] and overall or disease-specific survival in patients with RCC. **C** and **D** Western blot and dot plot analyses respectively revealed levels of cleaved VCAN (versikine) (**C**) and secretion of ADAMTS1 and versikine (**D**) in ADAMTS1-manipulated Caki-1 cells. (**E** and **F**) Transfection of VCAN-specific shRNA into ADAMTS1-overexpressing Caki-1 cells led to the collection of conditioned media and cell lysates for detecting secreted versikine (**E**) and the expressions or phosphorylation of ADAMTS1, EGFR, ERK, and Stat3 (**F**). (**I**) Dot plot demonstrated the correlation between the single sample gene set enrichment analysis (ssGSEA) score of “negative regulation of anoikis” and VCAN expression in TCGA-KIRC patients. A Pearson correlation was performed to evaluate their association and significance. **G, H, J** Cell viability (**G**), cell invasive ability (**H**), and in vivo metastatic potential (**J**) of Caki-1 cells expressing VCAN or EGFR shRNA with or without coexpression of ADAMTS1-flag were respectively assessed under suspended conditions with a CCK8 assay, Matrigel invasion assay, and zebrafish xenograft model. Values are presented as the mean ± SD of three independent experiments. In **G** and **J**, statistical analysis was performed using Student’s* t*-test. *p < 0.05, ***p < 0.001, compared with the control group; ^#^*p* < 0.05, ^###^*p* < 0.001, compared with the ADAMTS1-overexpressing only group. In **H**, data were analyzed using a one-way analysis of variance. Different letters represent various levels of significance. (**K**) High expression levels of both the *ADAMTS1* and *VCAN* genes were associated with the poorest overall survival in patients with RCC. The *p* value reflects a comparison between ADAMTS1^high^/VCAN^high^ and ADAMTS1^low^/VCAN^low^. The RCC dataset was obtained from TCGA

To further investigate the potential mechanisms underlying ADAMTS1-modulated anoikis resistance and invasive ability, we analyzed interacting neighbors of ADAMTS1 using the STRING database (Fig. 4A, left). Among the top ten interactors of ADAMTS1, we identified two proteoglycans of the ECM, versican (VCAN), and aggrecan (ACAN) (Fig. 4A, right). VCAN and ACAN are recognized as substrates of ADAMTS1, influencing cancer progression or metastasis [20, 27, 28]. As illustrated in Fig. 4B, a forest plot displays the hazard ratio of VCAN and ACAN associated with OS and DSS times in the ccRCC cohort from TCGA-KIRC dataset. We observed that elevated VCAN, but not ACAN, was significantly correlated with a worse prognosis in patients with ccRCC (Fig. 4B). Furthermore, significantly higher VCAN levels in tumor tissues compared with normal tissues were observed in different RCC cohorts obtained from the TNMplot and GEO (GSE105288, GSE46699, GSE159115) databases (Additional file 1: Supplementary Fig. 4A–C). Additionally, VCAN expression levels in RCC tissues were significantly correlated with advanced clinical stages (stages III + IV) and ADAMTS1 expression levels (Additional file 1: Supplementary Fig. 4D, E), suggesting that VCAN might be involved in ADAMTS1-induced RCC progression. We then identified which isoforms of VCAN were present in RCC cells through an RT–qPCR, revealing that VCAN V1 was expressed much more abundantly than VCAN V0 in both Caki-1 and 786-O cells. Neither VCAN V2 nor VCAN V3 were observed in either cell line (Additional file 1: Supplementary Fig. 5A). We subsequently analyzed ADAMTS1-cleaved VCAN (versikine) using anti-DPEAAE, a specific antibody that detects the neoepitope resulting from proteolysis of VCAN at the Glu^441^-Ala^442^ site (V1 sequence enumeration) [19]. Our observations indicated that overexpression and KD of ADAMTS1 respectively promoted and suppressed expression of 70-kDa cleaved VCAN in Caki-1 cells (Fig. 4C). Furthermore, secretion of cleaved VCAN also respectively increased and decreased in CM of Caki-1 cells (Fig. 4D). To further assess the importance of cleaved VCAN in ADAMTS1-induced EGFR activation, anoikis resistance, and cell invasion, we knocked down VCAN in ADAMTS1-overexpressing RCC cells. We found that VCAN-KD significantly rescued VCAN secretion (Fig. 4E, Additional file 1: Supplementary Fig. 5B), EGFR activation (Fig. 4F, Additional file 1: Supplementary Fig. 5C), anoikis resistance (Fig. 4G, Additional file 1: Supplementary Fig. 5D), and invasion promotion (Fig. 4H, Additional file 1: Supplementary Fig. 5E) induced by ADAMTS1 overexpression in Caki-1 and 786-O cells. Additionally, treatment of both cell lines with CM obtained from ADAMTS1-overexpressing cells also induced EGFR activation, while CM from RCC cells with ADAMTS1 overexpression and VCAN-KD failed to trigger EGFR activation (Additional file 1: Supplementary Fig. 5F). Importantly, we further utilized an additional shRNA for VCAN-KD and observed a reversal of the EGFR activation and anoikis resistance induced by ADAMTS1 overexpression in Caki-1 cells (Additional file 1: Supplementary Fig. 6). Clinically, we also observed that VCAN expression is positively correlated with ssGSEA score of “negative regulation of anoikis” pathway in TCGA-KIRC patients (Fig. 4I). Taken together, these results suggest that EGFR signaling may be triggered by ADAMTS1-mediated cleavage of VCAN, thereby promoting anoikis resistance and invasion by RCC cells. In vivo, we also observed a significant reversal of increased tumor metastasis in ADAMTS1-overexpressing Caki-1 cells when these cells were engineered to express shRNA targeting VCAN or the EGFR (Fig. 4J). In clinical analyses using the same TCGA-KIRC dataset, Kaplan–Meier curves revealed that patients with RCC with ADAMTS1^high^/VCAN^high^ tumors had significantly shorter survival times compared with patients with ADAMTS1^low^/VCAN^low^ tumors (Fig. 4K), suggesting the pivotal role of the ADAMTS1–VCAN–EGFR axis in RCC progression.

### The metalloproteinase activity of ADAMTS1 plays a crucial role in inducing the secretion of cleaved versican, EGFR activation, invasion, and anoikis resistance in RCC cells

**Fig. 5:**
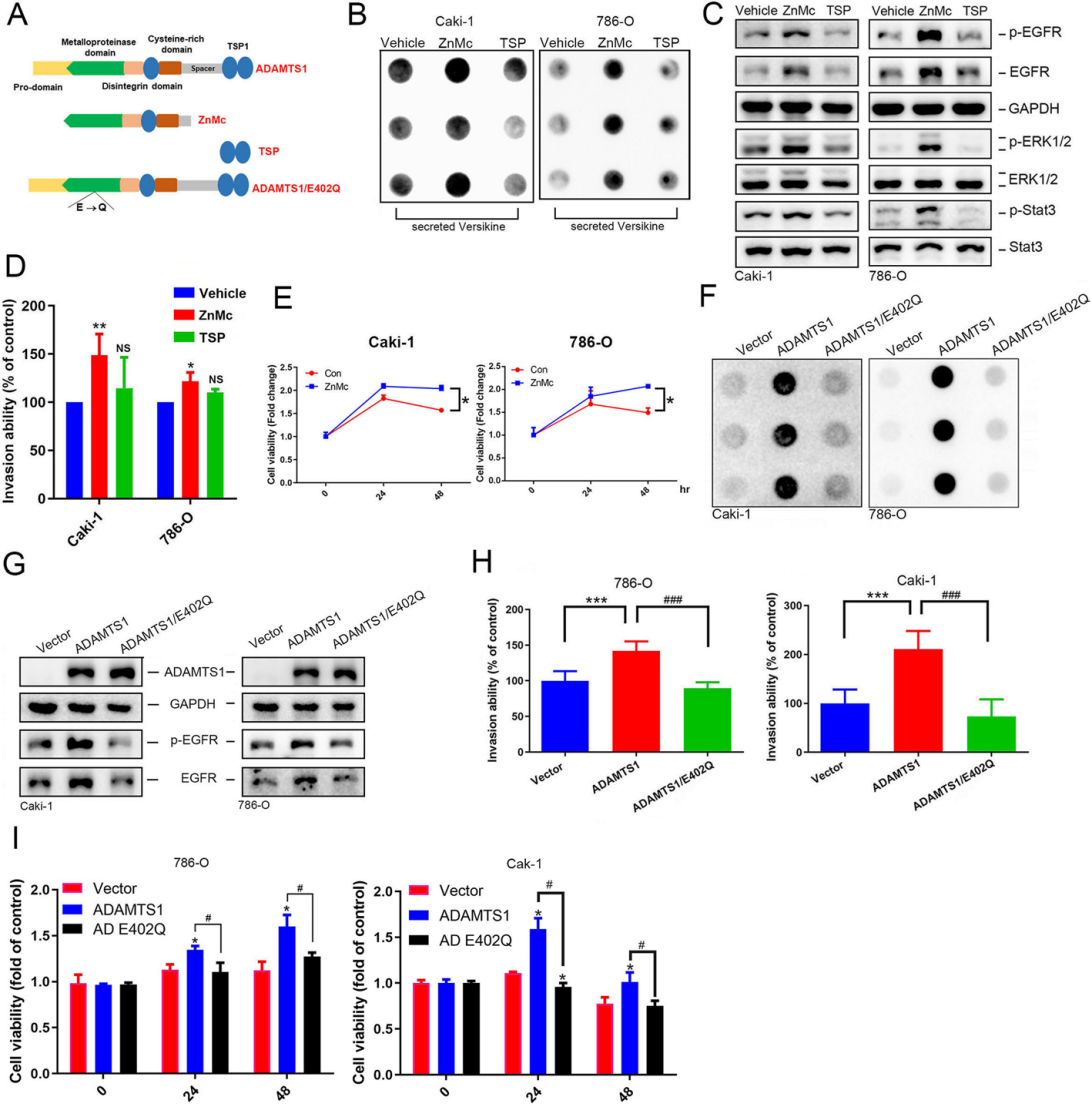
The metalloproteinase activity of ADAMTS1 is pivotal in stimulating the secretion of cleaved versican, activating the epidermal growth factor receptor (EGFR), promoting invasion, and conferring anoikis resistance in renal cell carcinoma (RCC) cells. **A** A schematic representation depicting two variants of the recombinant ADAMTS1 protein: one containing the metalloproteinase domain (ZnMc) and the other lacking it (TSP), along with a mutant ADAMTS1 expression construct (E402Q). **B**–**E** Treatment of Caki-1 cells with or without the recombinant ZnMc or TSP protein (40 nM) for 24 or 48 h. Subsequently, secretion of cleaved versican, activation of EGFR signal cascades, invasive abilities, and anoikis were respectively assessed using dot blot (**B**), western blotting (**C)**, Matrigel invasion (**D)**, and CCK8 assays. **F**–**I** Cleaved versican secretion (**F**), EGFR activation (**G**), invasive abilities (**H**), and anoikis (**I**) were evaluated in Caki-1 and 786-O cells after transducing wild-type (WT) ADAMTS1, ADAMTS1/E402Q, or a control vector. In **D**, **E**, **H**, and **I**, values are presented as the mean ± SD of three independent experiments. *p < 0.05, **p < 0.01, ***p < 0.001, compared with the control group; ^#^*p* < 0.05, ^###^*p* < 0.001, compared with the WT ADAMTS1-overexpressing group

The proteolytic cleavage of ADAMTS-1 requires its own metalloproteinase activity [29]. Herein, Caki-1 and 786-O cells were treated with two variants of the recombinant ADAMTS1 protein—one containing the metalloproteinase domain (ZnMc) and the other lacking it (TSP) (Fig. 5A). We observed that treatment with ZnMc but not TSP1 led to enhanced secretion of cleaved VCAN (Fig. 5B) and activation of EGFR signaling (Fig. 5C) in both cell lines. Functionally, only ZnMc treatment induced an increase in the invasive ability (Fig. 5D) and anoikis resistance (Fig. 5E) in both cell lines, suggesting that the metalloproteinase domain of ADAMTS1 is crucial for its role in mediating VCAN cleavage, EGFR activation, invasion, and anoikis resistance in RCC cells. To validate these findings, we generated a mutant form of ADAMTS1 by introducing a mutation (E402Q) in the metalloproteinase domain (Fig. 5A), rendering it inactive. Compared with the overexpression of wild-type ADAMTS1, overexpression of proteolytic inactive ADAMTS1 led to reductions in the secretion of cleaved VCAN (Fig. 5F), EGFR activation (Fig. 5G), invasive ability (Fig. 5H), and anoikis resistance (Fig. 5I) in RCC cell lines. Collectively, these findings suggest that metalloproteinase activity is indispensable for ADAMTS1-induced EGFR activation and subsequently promotes the metastasis of RCC cells.

### TIMP3 plays a critical role in controlling ADAMTS1 activity, thereby modulating versican cleavage, EGFR activation, and RCC progression

**Fig. 6:**
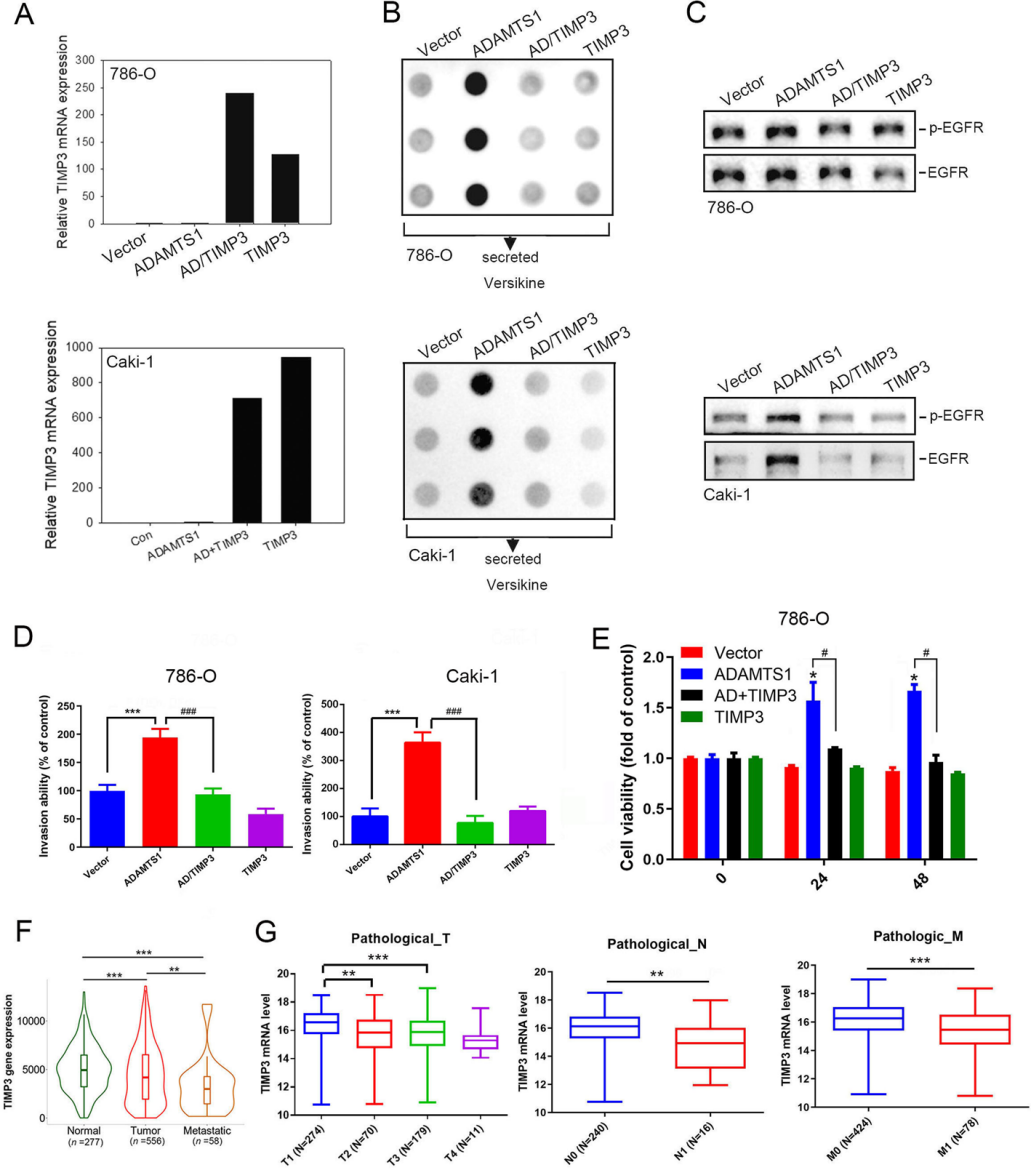
Tissue inhibitor of metalloproteinase 3 (TIMP3) expression reverses ADAMTS1-induced versican cleavage, epidermal growth factor receptor (EGFR) activation, and invasion and anoikis resistance of renal cell carcinoma (RCC) cells. **A** The pcDNA3-TIMP3 plasmid was transfected into ADAMTS1-overexpressing Caki-1 and 786-O cells as indicated, followed by measurement of TIMP3 mRNA levels using an RT–qPCR. Quantitative results of TIMP3 mRNA levels were normalized to GAPDH mRNA levels. **B**–**E** Evaluation of cleaved versican secretion **B**, EGFR activation **C**, invasive abilities **D**, and anoikis (**E** in RCC cells overexpressing ADAMTS1, subsequent to transfection with either a vector control or TIMP3 plasmid. In **D** and **E**, the values are presented as the mean ± SD of three independent experiments. *p < 0.05, ***p < 0.001, compared with the vector control group; ^#^*p* < 0.05, ^###^*p* < 0.001, compared with the ADAMTS1-overexpressing group. **F** Comparative analysis of TIMP3 mRNA levels among normal renal tissues, primary RCC, and metastatic RCC as determined by TNMplot. **G** Examination of *TIMP3* gene expression levels in RCC from TCGA based on the pathological tumor (T), node (N), and metastasis (M) stages

As shown in Fig. 4A, TIMP3 is another of the top ten interactors of ADAMTS1 and was reported to be the most potent inhibitor of ADAMTS1 metalloproteinase activity within the TIMP family [30]. To further investigate the role of TIMP3 expression in ADAMTS1-regulated cell invasive ability and anoikis resistance, we overexpressed TIMP3 in Caki-1 and 786-O cells with ADAMTS1 overexpression (Fig. 6A). Overexpression of TIMP3 markedly reversed the secretion of cleaved VCAN (Fig. 6B), EGFR activation (Fig. 6C), promotion of invasion (Fig. 6D), and resistance to anoikis (Fig. 6E) induced by ADAMTS1 overexpression in RCC cell lines. In a clinical setting, we observed significantly lower TIMP3 expression in RCC tissues, especially in metastatic RCC tissues, compared with normal kidney tissues (Fig. 6F). Additionally, TIMP3 expression levels in RCC tissues were inversely correlated with pathological TNM (Fig. 6G) and clinical stages (Additional file 1: Supplementary Fig. 7). These results suggest that TIMP3 acts as a tumor suppressor and a critical determinant for ATAMTS1–versican–EGFR cyclic axis-induced RCC progression.

### p53 is involved in ADAMTS1-modulated EGFR expression

**Fig. 7:**
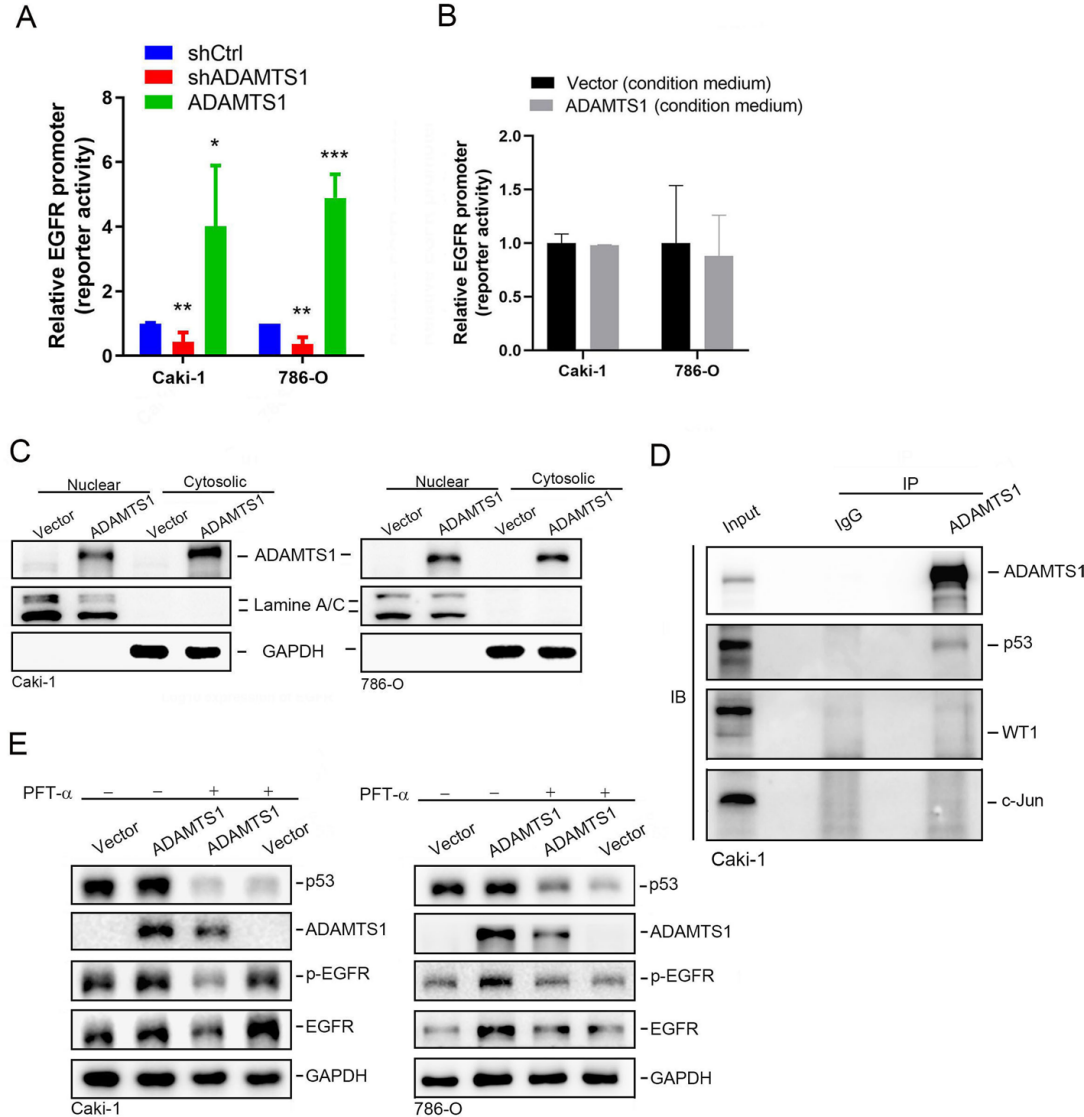
ADAMTS1 is associated with p53 to modulate epidermal growth factor receptor (EGFR) promoter activity and expression in renal cell carcinoma (RCC) cells. **A** Luciferase reporters were transfected into Caki-1 and 786-O cells with or without ADAMTS1 manipulation. All luciferase activity was normalized to Renilla reporter activity. The normalized reporter activity in cells transfected with a control vector was considered as onefold, and the relative fold change for each construct (ADAMTS1-flag or shADAMTS1) was further normalized to that of the control. **B** Reporters were transfected into Caki-1 and 786-O cells and treated with conditioned media (CM) collected from ADAMTS1-overexpressing or control Caki-1 and 786-O cells. The normalized reporter activity in cells treated with CM collected from control cells was considered onefold, and the relative fold change of RCC-derived CM treatment was calculated. The values are presented as the mean ± SD of three independent experiments. *p < 0.05, **p < 0.01, ***p < 0.001, compared with the control group. **C** Western blotting analysis of ADAMTS1 expression levels in cytoplasmic and nuclear fractions from Caki-1 and 786-O cells transfected with ADAMTS1-flag or a control vector. **D** The immunocomplex was precipitated from ADAMTS1-overexpressing Caki-1 cell lysates with an ADAMTS1 antibody and analyzed by western blotting to detect the associations of ADAMTS1 with indicated transcription factors (p53, WT1, and c-Jun). A normal IgG antibody was used as an immunoprecipitation (IP) control, and 10% whole-cell lysate was used as the input. **E** Western blot analysis of p53, ADAMTS1, p-EGFR, and EGFR levels in Caki-1 and 786-O cells after transducing ADAMTS1-flag or a control vector and treatment with the p53 inhibitor, pifithrin (PFT)-α (10 µM), or the vehicle for 24 h

In addition to EGFR transactivation induced by ADAMTS1, we observed downregulation and upregulation of EGFR mRNA in ADAMTS1-KD and ADAMTS1-overexpressing Caki-1 and 786-O cells, respectively (Fig. 3C, Additional file 1: Supplementary Fig. 2C). Furthermore, we respectively observed decreased and increased EGFR promoter activity when we knocked down and overexpressed ADAMTS1 in both cell lines (Fig. 7A), suggesting that ADAMTS1 can transcriptionally regulate EGFR in RCC cells. However, treatment of both cell lines with CM derived from ADAMTS1-overexpressing cells had no impact on EGFR promoter activity (Fig. 7B), indicating that expression of EGFR mRNA was not influenced by secreted ADAMTS1. Apart from extracellular ADAMTS1, the presence of ADAMTS1 in nuclei of breast cancer cells was reported [31]. Herein, we also observed that ADAMTS1 was present in nuclear fractions of ADAMTS1-overexpressing Caki-1 and 786-O cells (Fig. 7C). To further elucidate the role of nuclear ADAMTS1, we investigated whether ADAMTS1 was associated with transcription factors (TFs) located on the EGFR promoter. As shown in Fig. 7D and Additional file 1: Supplementary Fig. 8, p53 but not WT1 or c-Jun was present in the immunocomplex precipitated by an ADAMTS1-specific antibody in ADAMTS1-overexpressing Caki-1 and 786-O cells. p53 was reported to activate the EGFR promoter [32]. To confirm the importance of p53 in ADAMTS1-modulated total EGFR expression, we used the p53-specific inhibitor, pifithrin (PFT)-α. PFT-α treatment significantly downregulated p53 expression and reversed ADAMTS1 overexpression-induced increases in total EGFR and p-EGFR in Caki-1 and 786-O cells, suggesting that p53 is involved in ADAMTS1-modulated EGFR expression in RCC cells (Fig. 7E).

## Discussion

Anoikis resistance, linked to invasion and metastasis in various cancers [25, 33, 34], is a key target for antimetastatic therapies; yet, its molecular mechanisms in RCC remain poorly understood.

ADAMTS1 is one kind of ECM protease, which is implicated in a wide range of mechanistic pathways involved in cancer progression and metastasis of different cancer types [19]. In our present study, we identified the novel functional role of ADAMTS1 in promoting anoikis resistance, invasion, and metastasis of RCC cells in vitro and in vivo. Anoikis is a particular type of apoptosis induced by loss of cell adhesion to the ECM or inappropriate cell adhesion [35]. We observed that ADAMTS1 overexpression and KD in RCC cells respectively decreased and increased Bim, Bid, Bak, and PARP cleavage under suspension conditions. These results suggest that ADAMTS1 can suppress anoikis of detached RCC cells through blocking the mitochondrial pathway.

The proteoglycan VCAN is the most notable substrate of ADAMTS1 and was identified as a modulator of adhesion loss, cell motility, and tumor progression. VCAN can be cleaved by ADAMTS1 to release two fragments respectively containing the G1 and G3 domains [19]. The G1 fragment of VCAN was reported to regulate proapoptotic molecules, thus inhibiting the formation of the apoptotic mitochondrial machinery [36]. In our study, we found that only two of four variants of VCAN (V0 and V1) could be detected in RCC cells, and V1 was expressed at much higher levels than V0. ADAMTS1-mediated cleavage of V1 generated a small fragment of ~70 kDa consisting mainly of the G1 domain ending with the DPEAAE neoepitope at the C-terminus. VCAN G1-DPEAAE, also called versikine, actually increased in ADAMTS1-overexpressing RCC cells and decreased in ADAMTS1-KD RCC cells. Moreover, the secreted versikine level was also affected in ADAMTS1-manipulated RCC cells. Furthermore, KD of VCAN significantly reversed ADAMTS1-induced anoikis resistance of RCC cells. Taken together, ADAMTS1-modulated anoikis resistance of RCC cells may be dependent on VCAN V1 cleavage, which subsequently suppressed anoikis via blocking the mitochondrial pathway. Whether the death receptor pathway is also involved in ADAMTS1-mediated anoikis resistance needs to be further investigated in the future.

In addition to anoikis resistance, VCAN was reported to promote invasion of several cancer types, such as gastric and ovarian cancer cells [37, 38]. Herein, we observed that the ADAMTS1-induced invasion of RCC cells was significantly reversed by VCAN-KD, suggesting that VCAN also plays a critical role in the ADAMTS1-mediated invasive phenotype of RCC cells. From a zebrafish xenograft metastasis model, in fish that were inoculated with ADAMTS1-overexpressing Caki-1 cells, frequencies of fish showing trunk and end-tail dissemination dramatically increased compared with fish inoculated with control cells. We suggest that ADAMTS1-induced dissemination of cancer cells in vivo might be caused by VCAN-mediated invasion promotion and anoikis resistance.

Unregulated expression of growth factor receptors and their signaling pathways is linked to tumor malignancy due to hindrance of cell death pathways. Anomalous regulation of receptors like EGFR, TrkB, and HGFR activates prosurvival pathways such as PI3K/Akt, Ras/MAPK, and Rho-GTPase, fostering metastasis by inhibiting anoikis [11]. Results of the human p-RTK array revealed the downregulation of p-EGFR (p-ErbB1) in ADAMTS1-KD Caki-1 cells. To validate these findings, we conducted a western blot analysis, which demonstrated that ADAMTS1-KD and overexpression respectively suppressed and enhanced activation of EGFR and its downstream signals, including ERK and Stat3 in RCC cells. Total EGFR protein levels were also influenced in ADAMTS1-manipulated RCC cells. Moreover, EGFR-KD in ADAMTS1-overexpressing RCC cells significantly reversed heightened EGFR activation, anoikis resistance, and invasive ability. In in vivo experiments, EGFR-KD markedly reversed ADAMTS1-induced dissemination of cancer cells. Surprisingly, we observed that the ADAMTS1 level was also downregulated when EGFR was knocked down, suggesting that EGFR-activated signaling may exert positive feedback regulation on ADAMTS1 expression. In summary, our results suggest that cyclic escalation in the ADAMTS1–EGFR axis exacerbates anoikis resistance and invasive capabilities of RCC cells.

Previous research indicated that detachment from the ECM, leading to Bim expression, requires β1 integrin disengagement, downregulation of EGFR, and inhibition of ERK signalling [39], while ErbB2 overexpression impedes Bim expression and anoikis by upregulating α5 integrin and activating Src [40]. Additionally, ErbB2 overexpression was demonstrated to rescue both the EGFR and β1 integrin proteins through ERK and Sprouty2, thereby stabilizing the EGFR in ECM-detached cells [41]. Although our observations showed that ADAMTS1 can induce EGFR protein upregulation, Src and ERK activation, and anoikis resistance in RCC cells. Our p-RTK array also revealed downregulation of p-ErbB2 in ADAMTS1-KD Caki-1 cells (Additional file 1: Supplementary Fig. 9). A western blot analysis further confirmed that p-ErbB2 levels were downregulated in ADAMTS1-KD RCC cells (Additional file 1: Supplementary Fig. 9B). Further investigation is needed to determine whether ErbB2, Sprouty2, and integrin also play roles in ADAMTS1-modulated EGFR protein expression and anoikis resistance in RCC.

Regarding the interplay between ADAMTS1-mediated EGFR activation and VCAN cleavage, we observed that knocking-down VCAN significantly reversed the ADAMTS1 overexpression-induced phosphorylation of EGFR, ERK, and Stat3 in RCC cells. This suggests that VCAN cleavage plays a crucial role in ADAMTS1-mediated EGFR activation. Cleaved G3-VCAN, which contains two EGF-like repeats, was reported to activate the EGFR and stimulate proliferation through its EGF-like motifs [19]. It was demonstrated that brain tumor cells overexpressing a G3 mutant lacking two EGF-like motifs lost their characteristic of anchorage-independent growth [42]. These findings suggest that ADAMTS-1-mediated cleavage of VCAN generates cleaved G3-VCAN, further inducing EGFR-mediated anoikis resistance in RCC cells. Moreover, it was reported that integrin β1 binds to the G3 domain of VCAN [43], and integrins have the capability to form clusters with the EGFR, consequently influencing the intensity of EGFR-induced downstream signaling, such as ERK activation [44]. The involvement of integrins in ADAMTS1–VCAN axis-mediated EGFR activation, anoikis resistance, and invasion of RCC cells requires further investigation in future studies.

To further understand the functional aspects of ADAMTS1 in VCAN cleavage, invasion, and promotion of anoikis resistance, we observed that inactivating the metalloproteinase activity of ADAMTS1 with an E402Q mutation significantly reduced the enhanced VCAN cleavage, invasive ability, and anoikis resistance in RCC cells. This suggests that the catalytically active metalloproteinase domain of ADAMTS1 plays a critical role in ADAMTS1-induced tumor metastasis. It was reported that TIMP3 is the most potent endogenous inhibitor of ADAMTS1 metalloproteinase activity in the TIMP family [30]. Overexpression of TIMP3 can significantly reverse ADAMTS1-induced activation of the VCAN–EGFR axis, invasion, and anoikis resistance of RCC cells. Notably, we observed a reduction in TIMP3 expression in RCC tissues, especially in metastatic tumors, which was inversely correlated with the metastasis status of RCC. Similar to our findings, it was reported that most ccRCC cases exhibit loss of TIMP3, which was attributed to hypermethylation of the TIMP3 promoter [45]. Additionally, transforming growth factor (TGF)-β was demonstrated to induce TIMP3 expression, and strong correlations with expressions of TGFβRII and TIMP3 were observed in ccRCC specimens [45]. In esophageal cancer cells, loss of TGF-β was shown to activate ADAMTS1-mediated EGF-dependent invasion [46]. Taken together, the TGF-β–TGFβRII–TIMP3 axis may serve as a critical determinant in modulating RCC metastasis induced by the ADAMTS1–VCAN–EGFR axis.

Although ADAMTS1 was initially characterized as a secreted protease located in the ECM, recent findings by Silva at al. suggested that ADAMTS1 exhibits proteolytic activity within nuclei of breast cancer cells [31]. In our current investigation, we also observed nuclear expression of ADAMTS1 in RCC cells. Our study is the first to demonstrate that ADAMTS1 forms a complex with p53, thereby influencing EGFR expression and activation in Caki-1 and 786-O RCC cells. Notably, Caki-1 cells harbor wild-type p53, whereas 786-O cells possess mutations within the DNA-binding domain (DBD) of p53 [47]. It was previously reported that both wild-type and tumor-derived mutant p53 can activate the EGFR promoter [32]. Moreover, Ho at al. recently proposed that mutations in the DBD of p53 can sustain levels of p-EGFR in nuclei by disrupting the binding of the tyrosine phosphatase, SHP1 [48]. Investigating the underlying mechanism by which the ADAMTS1–p53 complex regulates EGFR expression and activation will be a focus of our future research endeavors.

## Conclusions

**Fig. 8:**
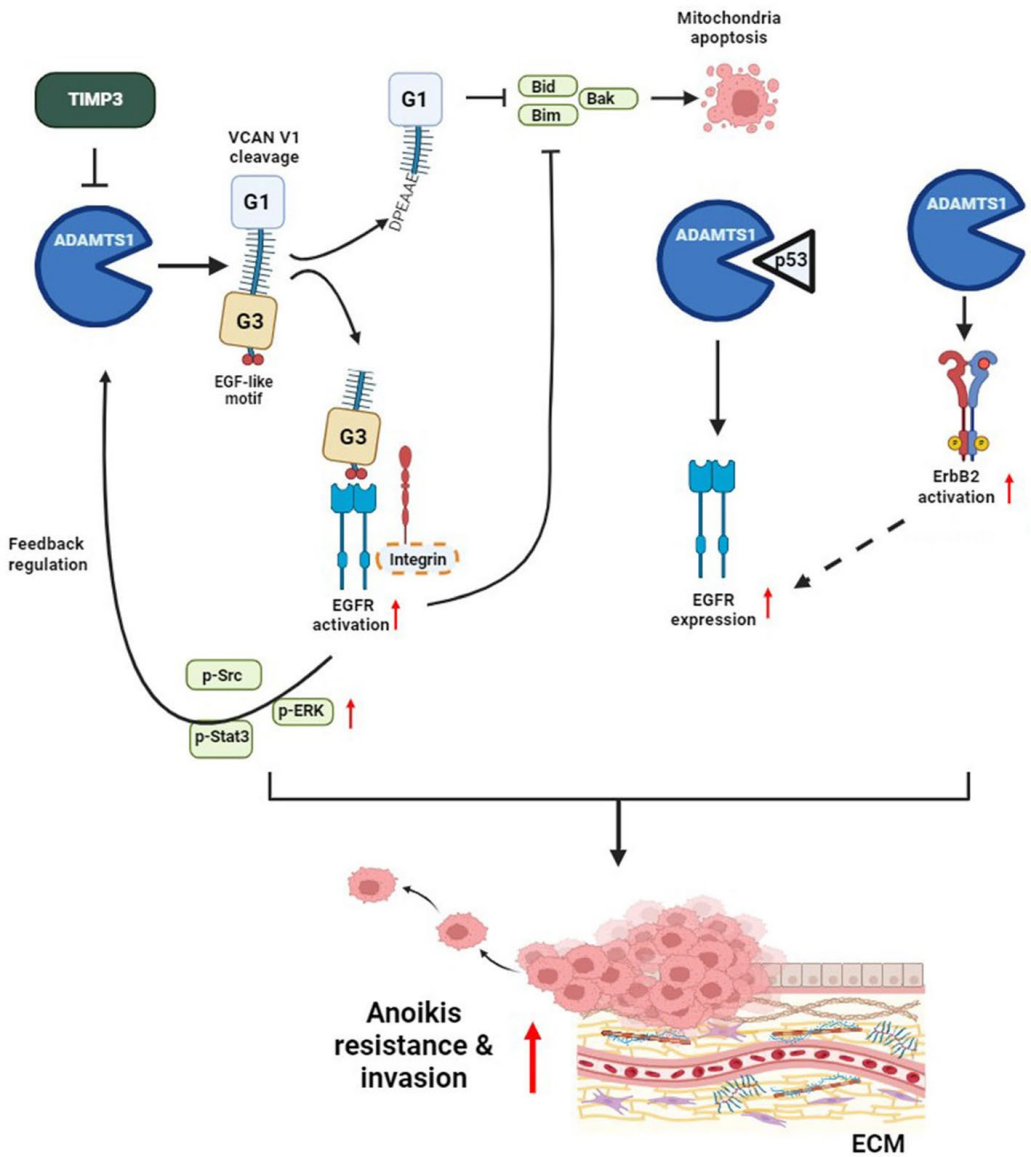
Schematic presentation depicting the ADAMTS1–VCAN–EGFR axis in promoting the anoikis resistance and invasive abilities of renal cell carcinoma (RCC). ADAMTS1 mediates the proteolytic cleavage of VCAN V1, thereby activating EGFR cascades to promote anoikis resistance and invasion of RCC cells. Moreover, ADAMTS1 interacts with p53 to modulate EGFR expression. EGFR-driven signaling might potentially reinforce ADAMTS1 expression through positive feedback. Bold dashed oval and arrow indicate hypothetical molecules and pathways that participate in ADAMTS1-mediated EGFR activation or expression. The cyclic elevation of ADAMTS1 and EGFR exacerbates anoikis resistance and invasiveness in RCC cells

In this study, we discovered a novel mechanism by which ADAMTS1 regulates tumor metastasis by inducing resistance to anoikis and invasion in RCC cells (Fig. 8). The ADAMTS1–EGFR cyclic axis serves as a promoter of tumor metastasis in RCC, with TIMP3, VCAN, and p53 being critical determinants involved in the execution of the prometastatic effect of the ADAMTS1–EGFR axis.

## Supplementary Information

Additional File 1.

Additional File 2.

## Data Availability

All data generated or analyzed in the current study are available from the corresponding author on reasonable request.

## Abbreviations

ACAN: Aggrecan
ADAMTS1: A disintegrin and metalloprotease with thrombospondin motifs 1
CM: Conditioned medium
Co-IP: Coimmunoprecipitation
DSS: Disease-specific survival
DBD: DNA-binding domain
ECM: Extracellular matrix
EGFR: Epidermal growth factor receptor
GEO: Gene Expression Omnibus
IP: Immunoprecipitation
KD: Knockdown
OS: Overall survival
MMP: Matrix metalloproteinase
RCC: Renal cell carcinoma
RTK: Receptor tyrosine kinase
TCGA-KIRC: The Cancer Genome Atlas Kidney Renal Clear Cell Carcinoma
TF: Transcription factor
TIMP3: Tissue inhibitor of metalloproteinase 3
TSP: Thrombospondin
VCAN: Versican
ZnMc: Zinc-dependent metalloprotease

## References

[1] Shuch B, Amin A, Armstrong AJ, Eble JN, Ficarra V, Lopez-Beltran A, at al. Understanding pathologic variants of renal cell carcinoma: distilling therapeutic opportunities from biologic complexity. Eur Urol. 2015;67(1):85–97. 24857407 10.1016/j.eururo.2014.04.029

[2] Sung WW, Ko PY, Chen WJ, Wang SC, Chen SL. Trends in the kidney cancer mortality-to-incidence ratios according to health care expenditures of 56 countries. Sci Rep. 2021;11(1):1479. 33446693 10.1038/s41598-020-79367-y PMC7809107

[3] Osawa T, Takeuchi A, Kojima T, Shinohara N, Eto M, Nishiyama H. Overview of current and future systemic therapy for metastatic renal cell carcinoma. Jpn J Clin Oncol. 2019;49(5):395–403. 30722031 10.1093/jjco/hyz013

[4] Kalra S, Atkinson BJ, Matrana MR, Matin SF, Wood CG, Karam JA, at al. Prognosis of patients with metastatic renal cell carcinoma and pancreatic metastases. BJU Int. 2016;117(5):761–5. 26032863 10.1111/bju.13185 PMC4666814

[5] Courtney KD, Choueiri TK. Updates on novel therapies for metastatic renal cell carcinoma. Ther Adv Med Oncol. 2010;2(3):209–19. 21789135 10.1177/1758834010361470 PMC3126014

[6] Tran J, Ornstein MC. Clinical review on the management of metastatic renal cell carcinoma. JCO Oncol Pract. 2022;18(3):187–96. 34529499 10.1200/OP.21.00419

[7] Serzan MT, Atkins MB. Current and emerging therapies for first line treatment of metastatic clear cell renal cell carcinoma. J Cancer Metastasis Treat. 2021. 10.2051/2394-4722.2021.76. 10.20517/2394-4722.2021.76 PMC8923624 35295921

[8] Paoli P, Giannoni E, Chiarugi P. Anoikis molecular pathways and its role in cancer progression. Biochim Biophys Acta. 2013;1833(12):3481–98. 23830918 10.1016/j.bbamcr.2013.06.026

[9] Gilmore AP. Anoikis. Cell Death Differ. 2005;12(Suppl 2):1473–7. 16247493 10.1038/sj.cdd.4401723

[10] Grossmann J. Molecular mechanisms of “detachment-induced apoptosis–Anoikis.” Apoptosis. 2002;7(3):247–60. 11997669 10.1023/a:1015312119693

[11] Kim YN, Koo KH, Sung JY, Yun UJ, Kim H. Anoikis resistance: an essential prerequisite for tumor metastasis. Int J Cell Biol. 2012;2012: 306879. 22505926 10.1155/2012/306879 PMC3296207

[12] Du S, Miao J, Zhu Z, Xu E, Shi L, Ai S, at al. NADPH oxidase 4 regulates anoikis resistance of gastric cancer cells through the generation of reactive oxygen species and the induction of EGFR. Cell Death Dis. 2018;9(10):948. 30237423 10.1038/s41419-018-0953-7 PMC6148243

[13] Guha D, Saha T, Bose S, Chakraborty S, Dhar S, Khan P, at al. Integrin-EGFR interaction regulates anoikis resistance in colon cancer cells. Apoptosis. 2019;24(11–12):958–71. 31641961 10.1007/s10495-019-01573-5

[14] Kim H, Sung JY, Park EK, Kho S, Koo KH, Park SY, at al. Regulation of anoikis resistance by NADPH oxidase 4 and epidermal growth factor receptor. Br J Cancer. 2017;116(3):370–81. 28081539 10.1038/bjc.2016.440 PMC5294491

[15] Peppicelli S, Ruzzolini J, Bianchini F, Andreucci E, Nediani C, Laurenzana A, at al. Anoikis resistance as a further trait of acidic-adapted melanoma cells. J Oncol. 2019;2019:8340926. 31275384 10.1155/2019/8340926 PMC6582804

[16] Nguyen BT, Lin CY, Chang TK, Fong YC, Thadevoos LA, Lai CY, at al. Melatonin inhibits chondrosarcoma cell proliferation and metastasis by enhancing miR-520f-3p production and suppressing MMP7 expression. J Pineal Res. 2023;75(1): e12872. 37057370 10.1111/jpi.12872

[17] Liu D, Zhang XX, Wan DY, Xi BX, Ma D, Wang H, at al. Sine oculis homeobox homolog 1 promotes α5β1-mediated invasive migration and metastasis of cervical cancer cells. Biochem Biophys Res Commun. 2014;446(2):549–54. 24613848 10.1016/j.bbrc.2014.03.002

[18] Takeuchi T, Adachi Y, Nagayama T, Furihata M. Matrix metalloproteinase-11 overexpressed in lobular carcinoma cells of the breast promotes anoikis resistance. Virchows Arch. 2011;459(3):291–7. 21773755 10.1007/s00428-011-1125-7

[19] Tan Ide A, Ricciardelli C, Russell DL. The metalloproteinase ADAMTS1: a comprehensive review of its role in tumorigenic and metastatic pathways. Int J Cancer. 2013;133(10):2263–76. 23444028 10.1002/ijc.28127

[20] Papadas A, Arauz G, Cicala A, Wiesner J, Asimakopoulos F. Versican and Versican-matrikines in Cancer Progression, Inflammation, and Immunity. J Histochem Cytochem. 2020;68(12):871–85. 32623942 10.1369/0022155420937098 PMC7711242

[21] Hirano T, Hirose K, Sakurai K, Makishima M, Sasaki K, Amano S. Inhibition of tumor growth by antibody to ADAMTS1 in mouse xenografts of breast cancer. Anticancer Res. 2011;31(11):3839–42. 22110207

[22] Cal S, López-Otín C. ADAMTS proteases and cancer. Matrix Biol. 2015;44–46:77–85. 10.1016/j.matbio.2015.01.013 25636539

[23] Bartha Á, Győrffy B. TNMplot.com: a web tool for the comparison of gene expression in normal, tumor and metastatic tissues. Int J Mol Sci. 2021;22:5. 10.3390/ijms22052622 PMC7961455 33807717

[24] Lin YW, Wen YC, Chu CY, Tung MC, Yang YC, Hua KT, at al. Stabilization of ADAM9 by N-α-acetyltransferase 10 protein contributes to promoting progression of androgen-independent prostate cancer. Cell Death Dis. 2020;11(7):591. 32719332 10.1038/s41419-020-02786-2 PMC7385149

[25] Banerjee D, Boboila S, Okochi S, Angelastro JM, Kadenhe-Chiweshe AV, Lopez G, at al. Activating transcription factor 5 promotes neuroblastoma metastasis by inducing anoikis resistance. Cancer Res Commun. 2023;3(12):2518–30. 38014922 10.1158/2767-9764.CRC-23-0154 PMC10714915

[26] Wen YC, Lin YW, Chu CY, Yang YC, Yang SF, Liu YF, at al. Melatonin-triggered post-transcriptional and post-translational modifications of ADAMTS1 coordinately retard tumorigenesis and metastasis of renal cell carcinoma. J Pineal Res. 2020;69(2): e12668. 32408377 10.1111/jpi.12668

[27] Asano K, Nelson CM, Nandadasa S, Aramaki-Hattori N, Lindner DJ, Alban T, at al. Stromal versican regulates tumor growth by promoting angiogenesis. Sci Rep. 2017;7(1):17225. 29222454 10.1038/s41598-017-17613-6 PMC5722896

[28] Lima MA, Dos Santos L, Turri JA, Nonogaki S, Buim M, Lima JF, at al. Prognostic value of ADAMTS proteases and their substrates in epithelial ovarian cancer. Pathobiology. 2016;83(6):316–26. 27359117 10.1159/000446244

[29] Kuno K, Terashima Y, Matsushima K. ADAMTS-1 is an active metalloproteinase associated with the extracellular matrix. J Biol Chem. 1999;274(26):18821–6. 10373500 10.1074/jbc.274.26.18821

[30] Minns AF, Qi Y, Yamamoto K, Lee K, Ahnström J, Santamaria S. The C-terminal domains of ADAMTS1 contain exosites involved in its proteoglycanase activity. J Biol Chem. 2023;299(4): 103048. 36813235 10.1016/j.jbc.2023.103048 PMC10033314

[31] Silva SV, Lima MA, Cella N, Jaeger RG, Freitas VM. ADAMTS-1 is found in the nuclei of normal and tumoral breast cells. PLoS ONE. 2016;11(10): e0165061. 27764205 10.1371/journal.pone.0165061 PMC5072708

[32] Ludes-Meyers JH, Subler MA, Shivakumar CV, Munoz RM, Jiang P, Bigger JE, at al. Transcriptional activation of the human epidermal growth factor receptor promoter by human p53. Mol Cell Biol. 1996;16(11):6009–19. 8887630 10.1128/mcb.16.11.6009 PMC231603

[33] Dai Y, Zhang X, Ou Y, Zou L, Zhang D, Yang Q, at al. Anoikis resistance–protagonists of breast cancer cells survive and metastasize after ECM detachment. Cell Commun Signal. 2023;21(1):190. 37537585 10.1186/s12964-023-01183-4 PMC10399053

[34] Wang J, Luo Z, Lin L, Sui X, Yu L, Xu C, at al. Anoikis-Associated Lung Cancer Metastasis Mechanisms and Therapies. Cancers. 2022;14:19. 10.3390/cancers14194791 PMC9564242 36230714

[35] Frisch SM, Francis H. Disruption of epithelial cell-matrix interactions induces apoptosis. J Cell Biol. 1994;124(4):619–26. 8106557 10.1083/jcb.124.4.619 PMC2119917

[36] Sheng W, Wang G, Wang Y, Liang J, Wen J, Zheng PS, at al. The roles of versican V1 and V2 isoforms in cell proliferation and apoptosis. Mol Biol Cell. 2005;16(3):1330–40. 15635104 10.1091/mbc.E04-04-0295 PMC551496

[37] Zhai L, Chen W, Cui B, Yu B, Wang Y, Liu H. Overexpressed versican promoted cell multiplication, migration and invasion in gastric cancer. Tissue Cell. 2021;73: 101611. 34358918 10.1016/j.tice.2021.101611

[38] Ween MP, Hummitzsch K, Rodgers RJ, Oehler MK, Ricciardelli C. Versican induces a pro-metastatic ovarian cancer cell behavior which can be inhibited by small hyaluronan oligosaccharides. Clin Exp Metastasis. 2011;28(2):113–25. 21153687 10.1007/s10585-010-9363-7

[39] Reginato MJ, Mills KR, Paulus JK, Lynch DK, Sgroi DC, Debnath J, at al. Integrins and EGFR coordinately regulate the pro-apoptotic protein Bim to prevent anoikis. Nat Cell Biol. 2003;5(8):733–40. 12844146 10.1038/ncb1026

[40] Haenssen KK, Caldwell SA, Shahriari KS, Jackson SR, Whelan KA, Klein-Szanto AJ, at al. ErbB2 requires integrin alpha5 for anoikis resistance via Src regulation of receptor activity in human mammary epithelial cells. J Cell Sci. 2010;123(Pt 8):1373–82. 20332114 10.1242/jcs.050906 PMC3705932

[41] Grassian AR, Schafer ZT, Brugge JS. ErbB2 stabilizes epidermal growth factor receptor (EGFR) expression via Erk and Sprouty2 in extracellular matrix-detached cells. J Biol Chem. 2011;286(1):79–90. 20956544 10.1074/jbc.M110.169821 PMC3013038

[42] Wu Y, Chen L, Cao L, Sheng W, Yang BB. Overexpression of the C-terminal PG-M/versican domain impairs growth of tumor cells by intervening in the interaction between epidermal growth factor receptor and beta1-integrin. J Cell Sci. 2004;117(Pt 11):2227–37. 15126624 10.1242/jcs.01057

[43] Wu Y, Chen L, Zheng PS, Yang BB. beta 1-Integrin-mediated glioma cell adhesion and free radical-induced apoptosis are regulated by binding to a C-terminal domain of PG-M/versican. J Biol Chem. 2002;277(14):12294–301. 11805102 10.1074/jbc.M110748200

[44] Yamada KM, Even-Ram S. Integrin regulation of growth factor receptors. Nat Cell Biol. 2002;4(4):E75–6. 11944037 10.1038/ncb0402-e75

[45] Masson D, Rioux-Leclercq N, Fergelot P, Jouan F, Mottier S, Théoleyre S, at al. Loss of expression of TIMP3 in clear cell renal cell carcinoma. Eur J Cancer. 2010;46(8):1430–7. 20194016 10.1016/j.ejca.2010.01.009

[46] Le Bras GF, Taylor C, Koumangoye RB, Revetta F, Loomans HA, Andl CD. TGFβ loss activates ADAMTS-1-mediated EGF-dependent invasion in a model of esophageal cell invasion. Exp Cell Res. 2015;330(1):29–42. 25064463 10.1016/j.yexcr.2014.07.021 PMC4267897

[47] Leroy B, Girard L, Hollestelle A, Minna JD, Gazdar AF, Soussi T. Analysis of TP53 mutation status in human cancer cell lines: a reassessment. Hum Mutat. 2014;35(6):756–65. 24700732 10.1002/humu.22556 PMC4451114

[48] Ho TLF, Lee MY, Goh HC, Ng GYN, Lee JJH, Kannan S, at al. Domain-specific p53 mutants activate EGFR by distinct mechanisms exposing tissue-independent therapeutic vulnerabilities. Nat Commun. 2023;14(1):1726. 36977662 10.1038/s41467-023-37223-3 PMC10050071

