# Supplementary material for: The oncogenic ADAMTS1–VCAN–EGFR cyclic axis drives anoikis resistance and invasion in renal cell carcinoma
Source: Cell Mol Biol Lett. 2024 Sep 27;29:126. doi: 10.1186/s11658-024-00643-0 (PMC11429190; doi:10.1186/s11658-024-00643-0)
Supplement: Supplementary file 1 — Additional File 1. [file 11658_2024_643_MOESM1_ESM.docx]

**Supplemental Information**

**Title:**

**The oncogenic ADAMTS1-VCAN-EGFR cyclic axis drives anoikis resistance and invasion in renal cell carcinoma**

Yu-Ching Wen, Yung-Wei Lin, Kuo-Hao Ho, Yi-Chieh Yang, Feng-Ru Lai, Chih-Ying Chu, Ji-Qing Chen, Wei-Jiunn Lee*, and Ming-Hsien Chien*

Correspondence to: Dr. Wei-Jiunn Lee (E-mail: lwj5905@gmail.com) and Dr. Ming-Hsien Chien (E-mail: mhchien1976@gmail.com)

**Figure Legends**

**
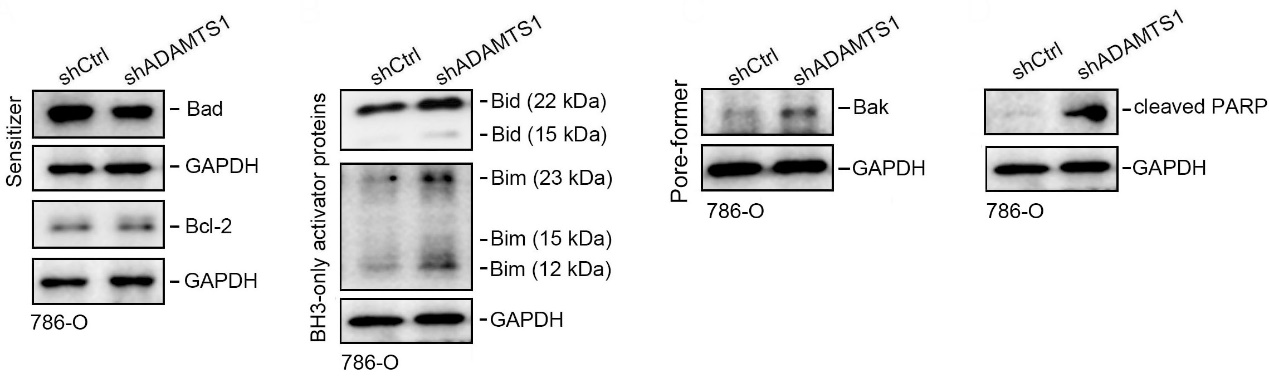
**

**Supplementary Fig. 1. Knockdown of ADAMTS1 increases expressions of Bid, Bim, Bak, and cleaved poly(ADP ribose) polymerase (PARP) in 786-O cells.** A Western blot analysis was conducted to assess expression levels of intrinsic apoptosis-related proteins (Bad, Bcl-2, Bak, Bid, Bim, and PARP) in suspended 786-O cells following knockdown of ADAMTS1. GAPDH was used as a loading control.


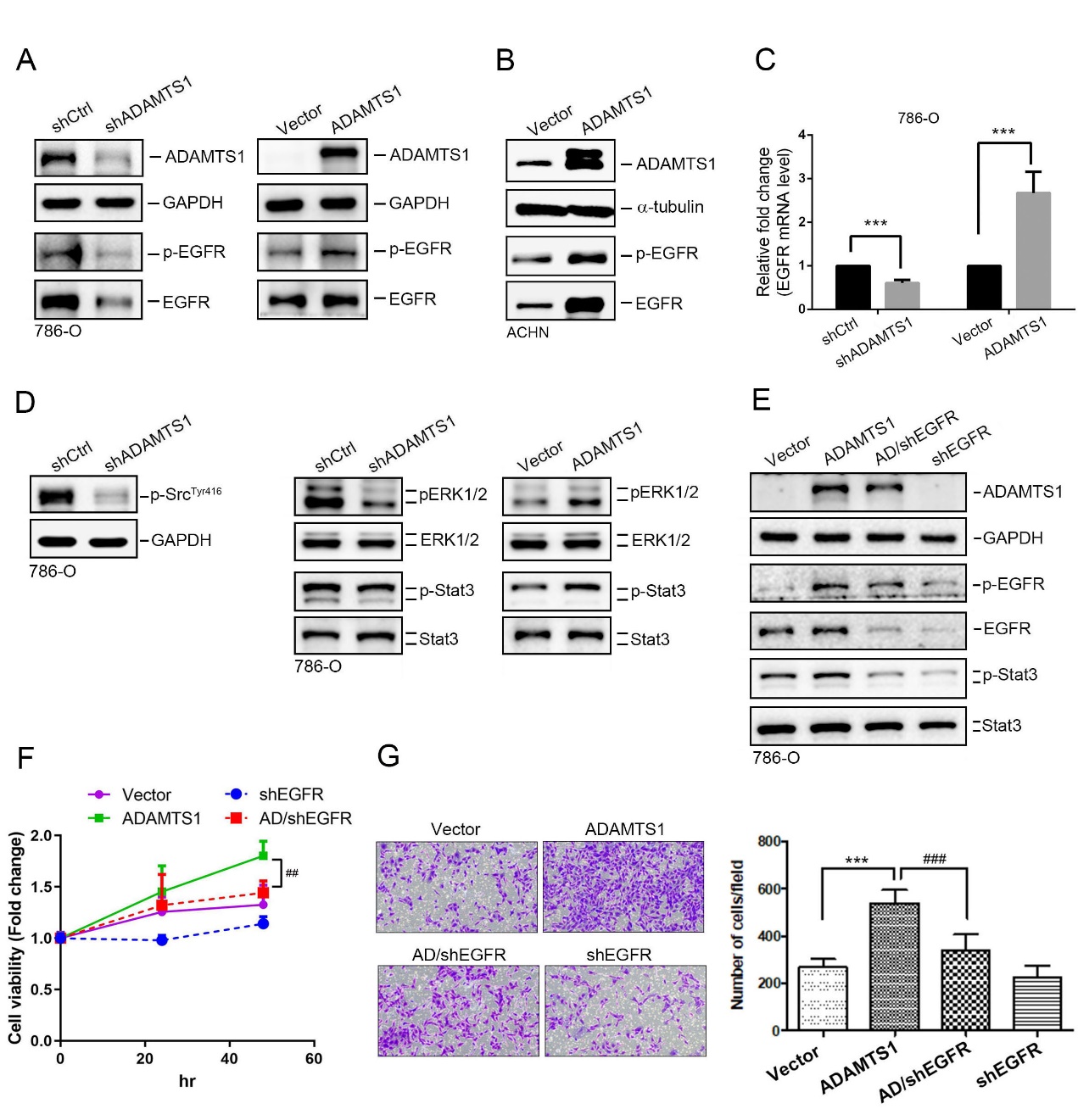


**Supplementary Fig. 2. ADAMTS1 expression activates epidermal growth factor receptor (EGFR) signaling cascades, leading to anoikis resistance and invasion by renal cell carcinoma (RCC) cells.** (A and B) 786-O (A) and ACHN cells (B) underwent knockdown or overexpression of ADAMTS1 to examine protein levels of phosphorylated (p)-EGFR and EGFR. (C and D) mRNA levels of EGFR and its downstream signaling cascades in ADAMTS1-manipulated 786-O cells were analyzed using real-time qPCR (C) and Western blotting (WB) assays (D). (E-G) EGFR shRNA was transfected into ADAMTS1-overexpressing 786-O cells as indicated, and the expressions or phosphorylation of ADAMTS1, EGFR, and Stat3 were assessed by WB (E). Additionally, cell viability in suspended conditions (F) and the cell invasive ability (G) were respectively evaluated with CCK8 and Matrigel invasion assays. Multiples of differences are presented as the mean ± SD of three independent experiments. *** *p* < 0.001, compared to the control group; ^##^ *p* < 0.01, ^###^ *p* < 0.001, compared to the ADAMTS1-overexpressing only group.


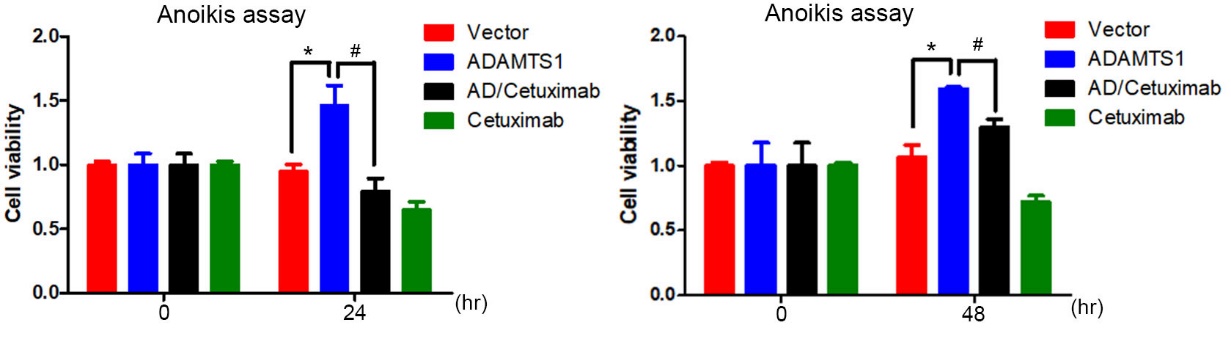


**Supplementary Fig. 3. Blocking epidermal growth factor receptor (EGFR) signaling cascades abolishes ADAMTS1-induced anoikis resistance in Caki-1 cells.** ADAMTS1-overexpressing cells were treated with 5 µg/ml cetuximab for the indicated time points. Cell viability under suspended conditions was assessed using CCK8 assays. * *p* < 0.05, compared to the control group; ^#^*p* < 0.05 compared to the ADAMTS1-overexpressing only group.


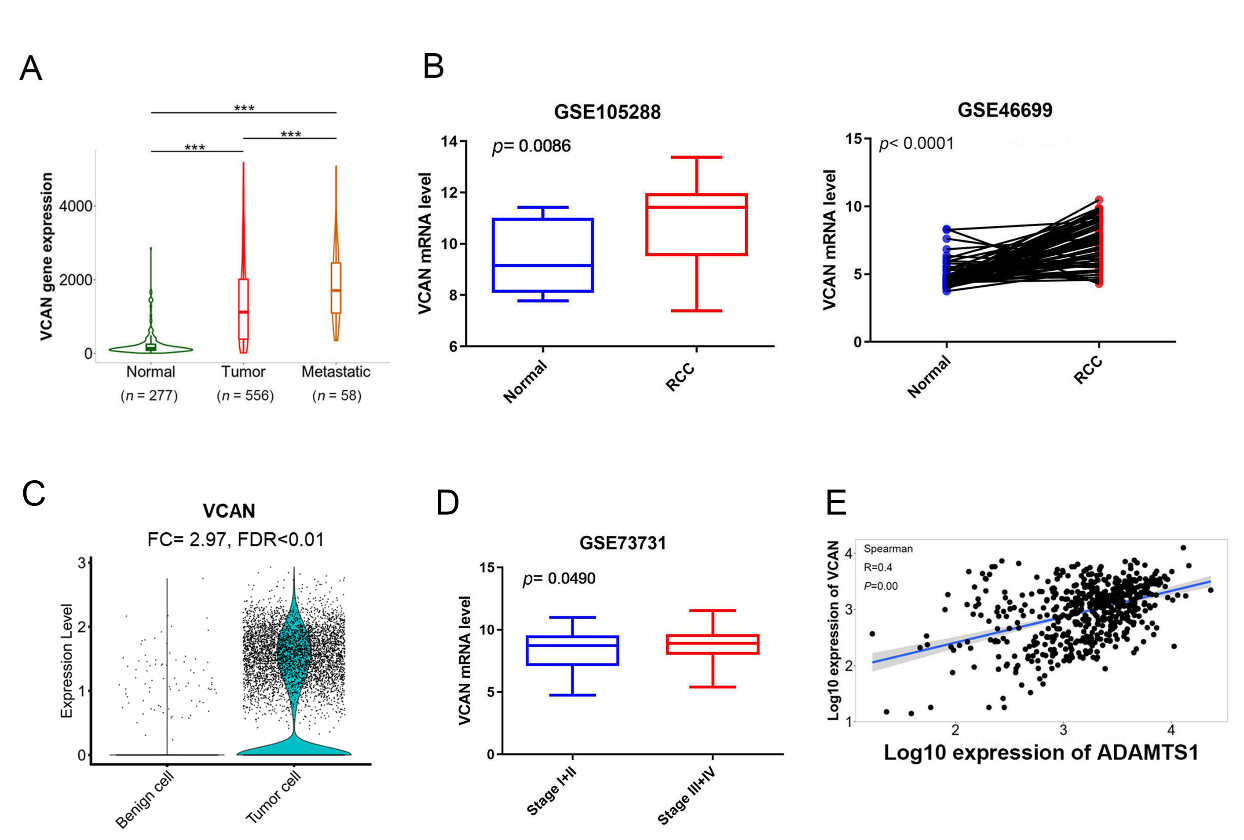


**Supplementary Fig. 4. Elevated versican (VCAN) expression in renal cell carcinoma (RCC) tissues is correlated with advanced clinical stages and ADAMTS1 expression.** (A) Comparative analysis of VCAN mRNA levels among normal renal tissues, primary RCC, and metastatic RCC as determined by TNMplot. (B) mRNA expression of VCAN in paired adjacent (GSE46699; right panel) and unpaired (GSE105288; left panel) normal and tumor tissues derived from patients with RCC. (C) The violin plot indicates that VCAN expression is significantly higher in tumor cells compared to benign epithelial cells, with a fold change (FC) of 2.97 and a false discovery rate (FDR) of less than 0.01. (D) VCAN expression levels in RCC from GSE73731 were compared according to clinical stages. (E) RCC dataset from TNMplot showed a positive correlation between ADAMTS1 and VCAN expression.


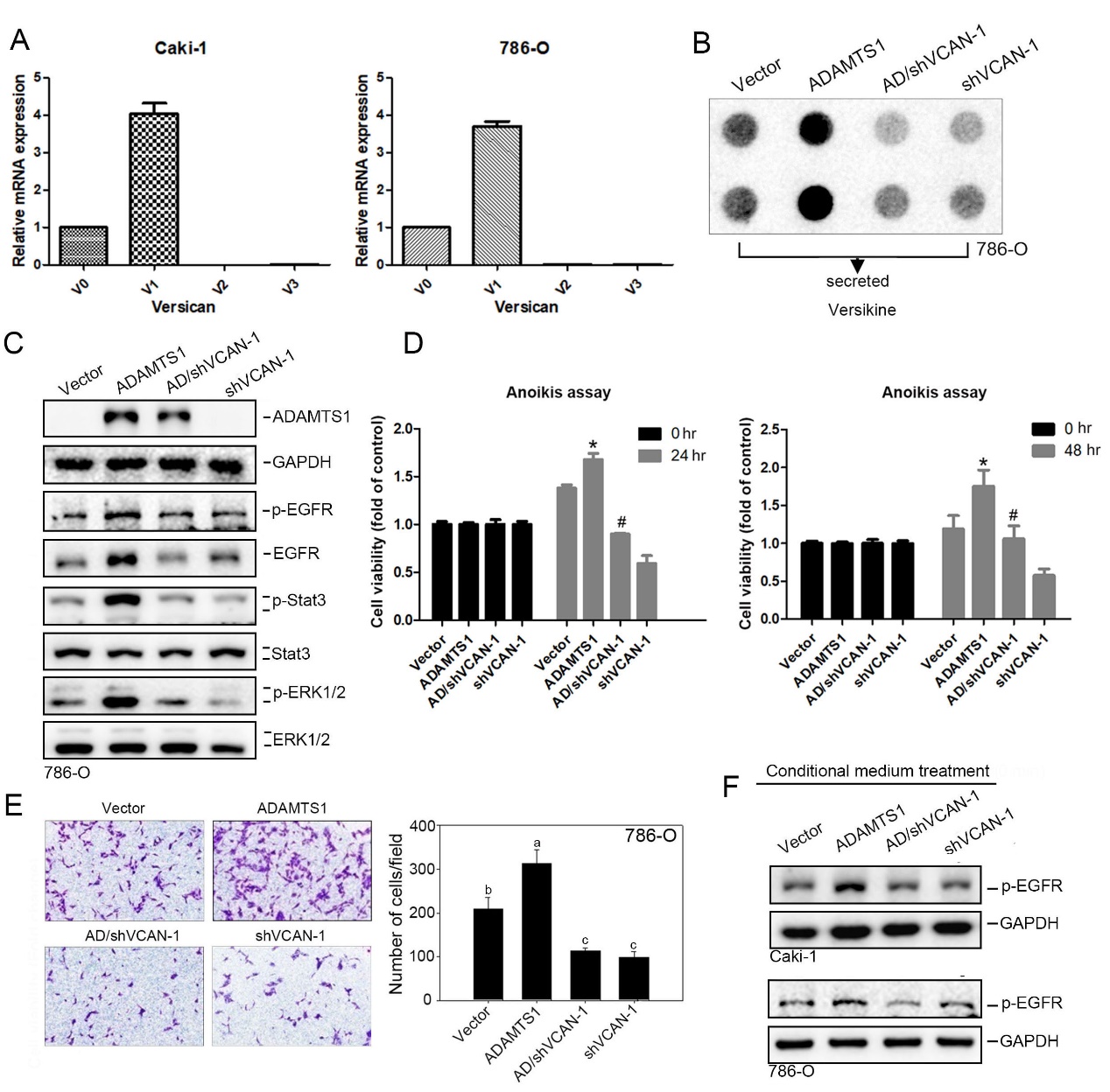


**Supplementary Fig. 5. The versican (VCAN) V1 isoform exhibits the highest expression in renal cell carcinoma (RCC) cells and is cleaved by ADAMTS1, thereby triggering epidermal growth factor receptor (EGFR) activation, conferring anoikis resistance, and promoting invasion by 786-O RCC cells.** (A) A real-time qPCR was used to detect levels of VCAN isoforms (V1~V4) in both 786-O and Caki-1 cells. (B and C) Transfection of a VCAN-specific shRNA into ADAMTS1-overexpressing 786-O cells resulted in the collection of conditioned media (CM) and cell lysates to detect secreted versikine (B) and the expression or phosphorylation of ADAMTS1, EGFR, extracellular signal-regulated kinase (ERK), and signal transduction and activator of transcription 3 (Stat3) (C). (D and E) 786-O cells expressing VCAN shRNA with or without co-expression of ADAMTS1-flag were subjected to assessment of cell viability under suspended conditions (D) and invasive ability (E) respectively using CCK8 and Matrigel invasion assays. In (D), statistical analysis was performed using Student’s *t*-test. * *p* < 0.05, compared to the control group; ^#^ *p* < 0.05, compared to the ADAMTS1-overexpressing only group. In E, data were analyzed using a one-way ANOVA. Different letters represent varying levels of significance. (F) Treatment of CM from Caki-1 and 786-O cells with manipulation of the *ADAMTS1* and *VCAN* genes as indicated, and EGFR phosphorylation levels in both cell lines were determined.


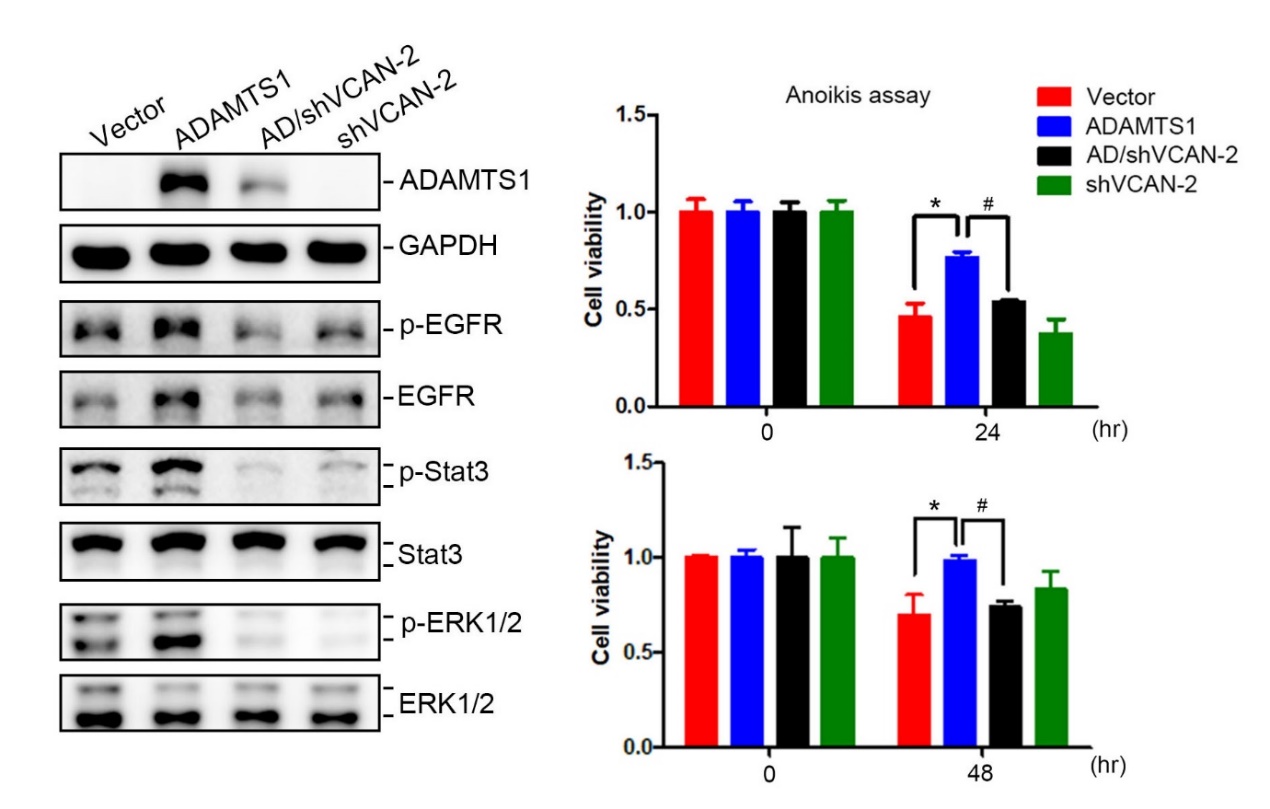


**Supplementary Fig. 6. The expression of versican (VCAN) is necessary for ADAMTS1-induced epidermal growth factor receptor (EGFR) activation and anoikis resistance in Caki-1 RCC cells.** Transfection of another VCAN-specific shRNA into ADAMTS1-overexpressing Caki-1 cells and collected cell lysates to detect the expression or phosphorylation of ADAMTS1, EGFR, ERK, and Stat3. Cells expressing VCAN shRNA with or without ADAMTS1-overexpression were subjected to assessment of cell viability at indicated time points under suspended conditions using CCK8 assays. * *p* < 0.05, compared to the control group; ^#^ *p* < 0.05, compared to the ADAMTS1-overexpressing only group.


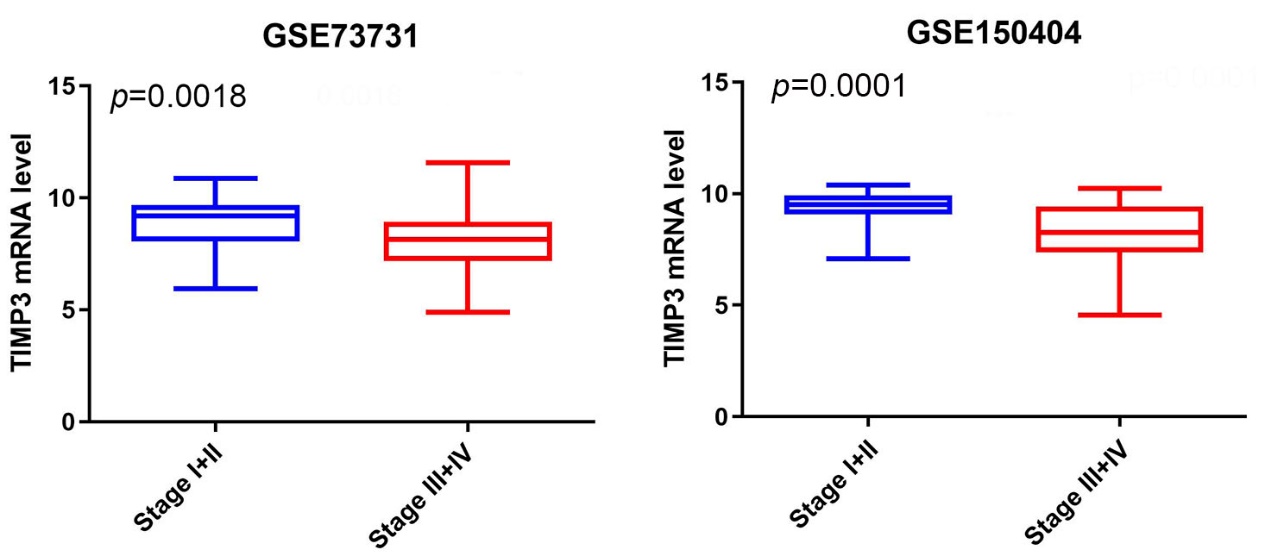


**Supplementary Fig. 7.** **Tissue inhibitor of metalloproteinase 3 (TIMP3) expression is inversely correlated with clinical stages in patients with renal cell carcinoma (RCC).** TIMP3 expression levels in different RCC cohorts obtained from the GSE73731 and GSE150404 datasets were compared based on clinical stages.


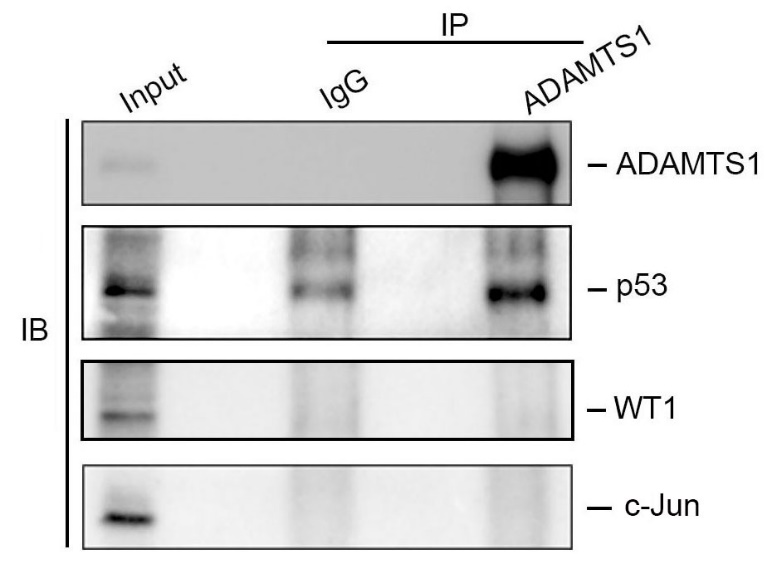


**Supplementary Fig. 8.** **ADAMTS1 forms a complex with p53 in 786-O renal cell carcinoma (RCC) cells.** The immunocomplex was precipitated from lysates of ADAMTS1-overexpressing 786-O cells using an ADAMTS1 antibody, and then subjected to Western blotting to examine interactions of ADAMTS1 with specified transcription factors (p53, WT1, and c-Jun). A normal IgG antibody served as a control for immunoprecipitation (IP), and 10% whole-cell lysates were utilized as input.


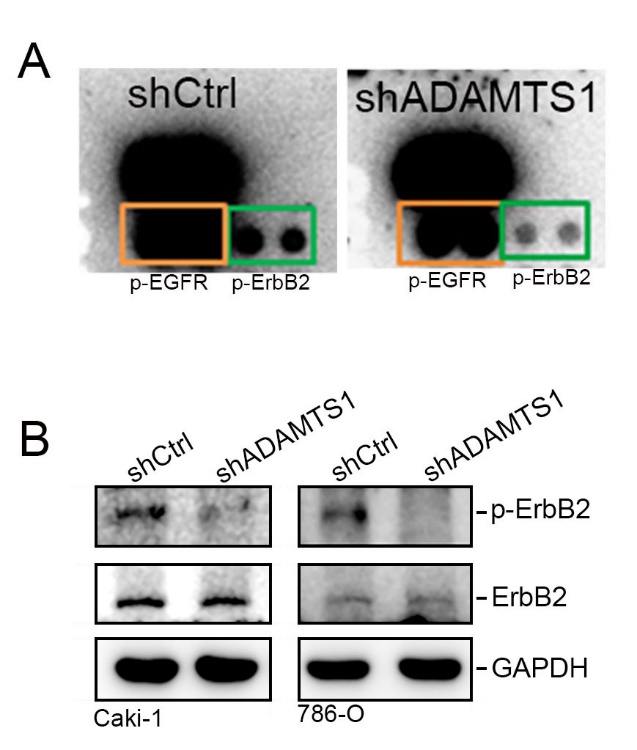


**Supplementary Fig. 9.** **Knockdown of ADAMTS1 induces downregulation of phosphorylated ErbB2 (p-ErbB2) and ErbB2 in renal cell carcinoma (RCC) cells.** (A) Screening with a human receptor tyrosine kinase (RTK) array indicates downregulation of p-ErbB2 in Caki-1 cells following ADAMTS1-knockdown compared to control cells. (B) Caki-1 and 786-O cells were subjected to knockdown of ADAMTS1 to assess protein levels of p-ErbB2 and ErbB2 using Western blotting.
