# Supplementary material for: The oncogenic ADAMTS1–VCAN–EGFR cyclic axis drives anoikis resistance and invasion in renal cell carcinoma
Source: Cell Mol Biol Lett. 2024 Sep 27;29:126. doi: 10.1186/s11658-024-00643-0 (PMC11429190; doi:10.1186/s11658-024-00643-0)

Fig. 2A

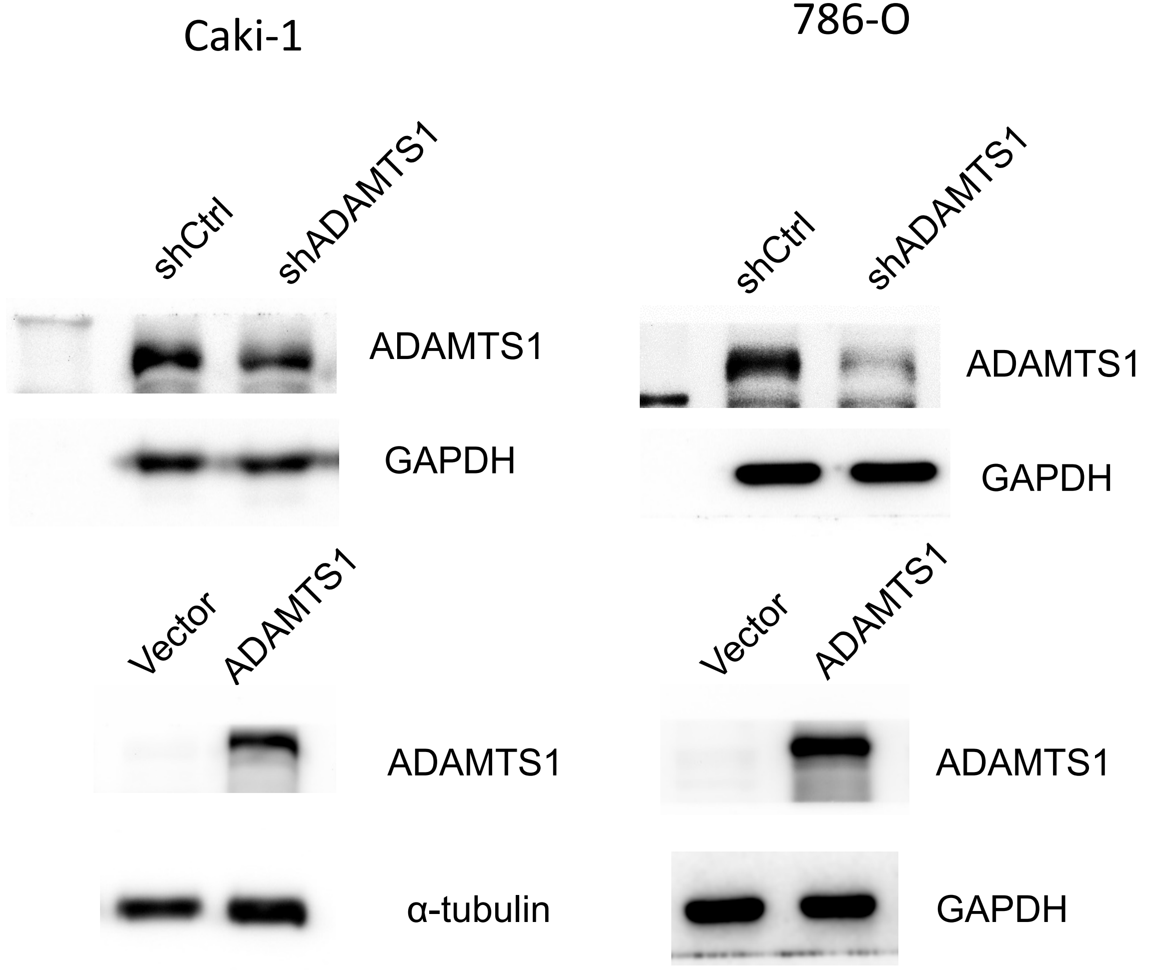
Caki-1
786-O
shCtrl
shADAMTS1
ADAMTS1
GAPDH
shCtrl
shADAMTS1
ADAMTS1
GAPDH
Vector
ADAMTS1
ADAMTS1
α-tubulin
Vector
ADAMTS1
ADAMTS1
GAPDH

Fig.2C-upper panel

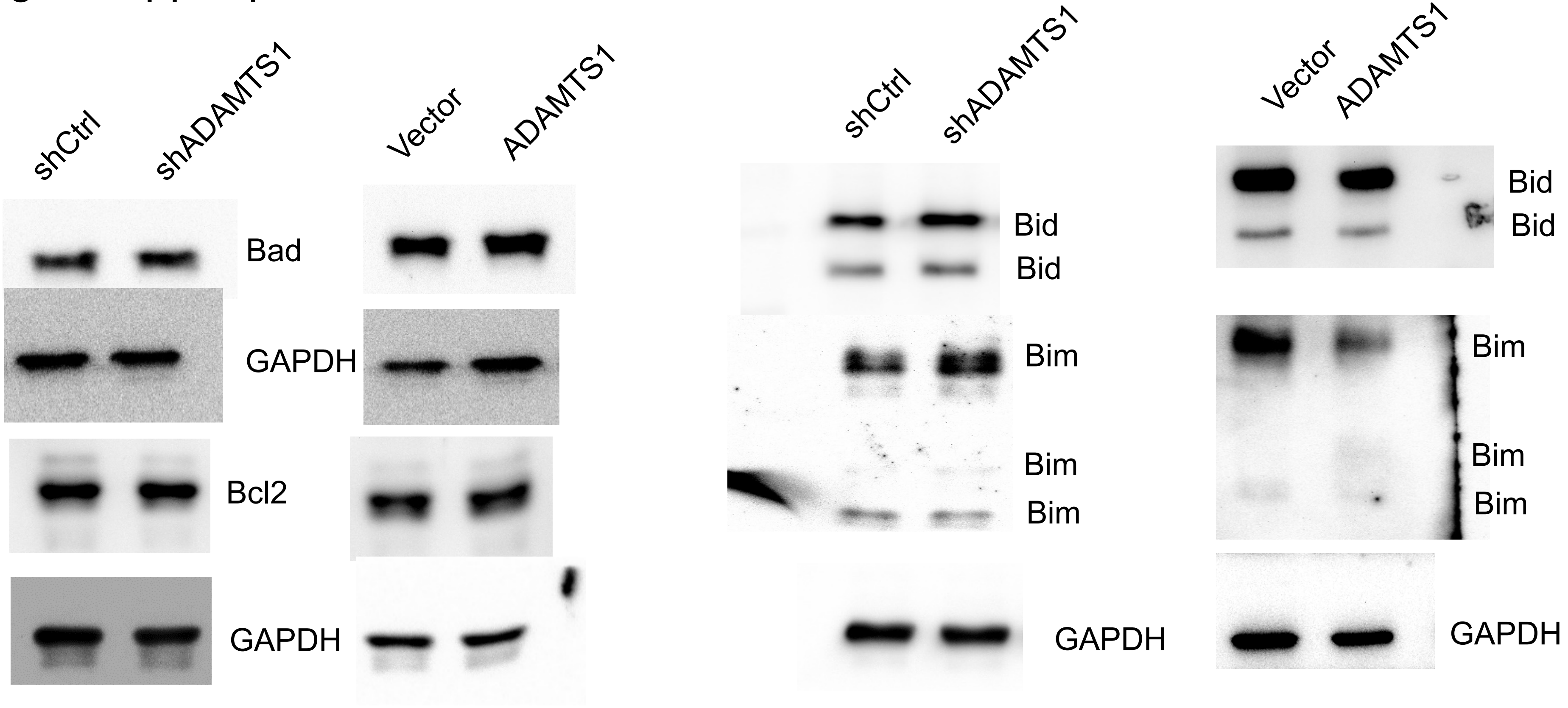

Fig.2C-lower panel

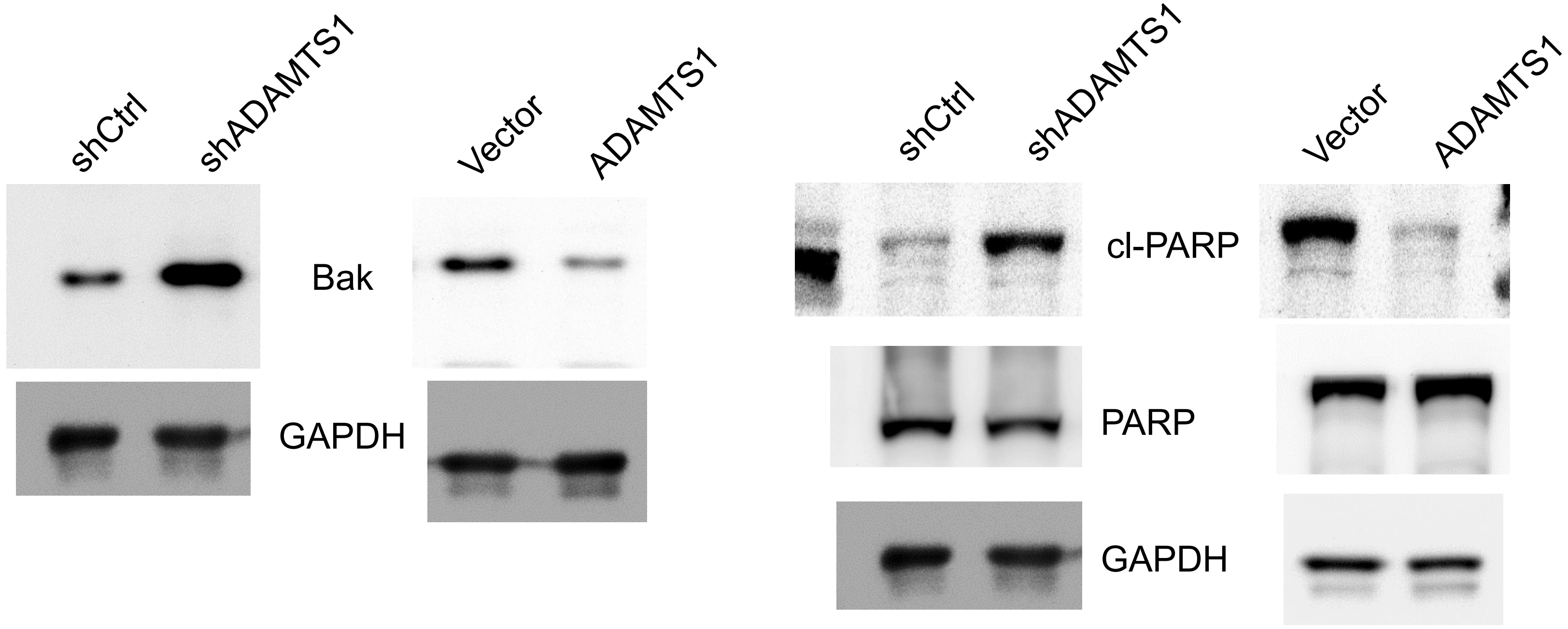

Fig.3B

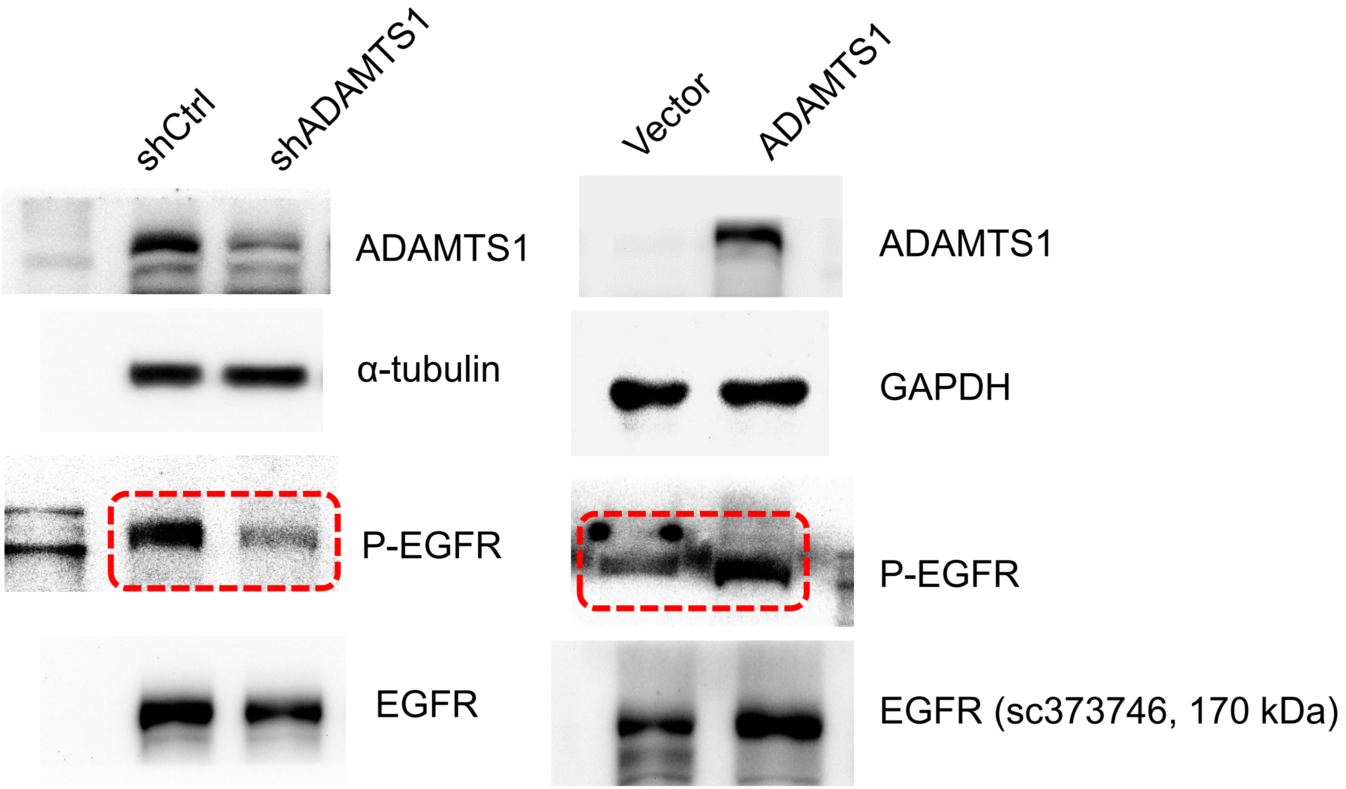

shCtrl
shADAMTS1
Vector
ADAMTS1
ADAMTS1
ADAMTS1
α-tubulin
GAPDH
P-EGFR
P-EGFR
EGFR
EGFR (sc373746, 170 kDa)

Fig.3D

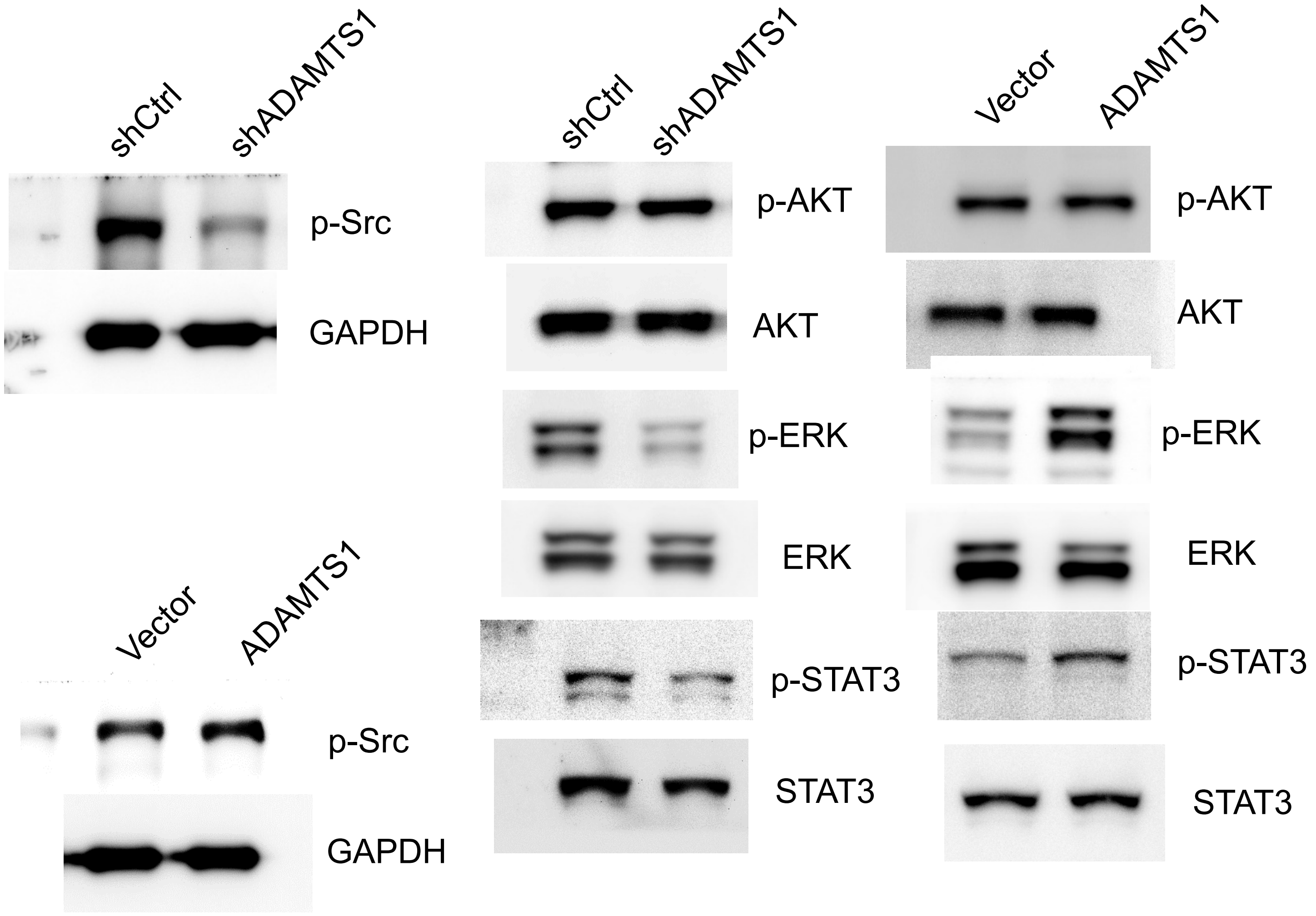
shCtrl
shADAMTS1
p-Src
GAPDH
Vector
ADAMTS1
p-Src
GAPDH
shCtrl
shADAMTS1
p-AKT
AKT
p-ERK
ERK
p-STAT3
STAT3
Vector
ADAMTS1
p-AKT
AKT
p-ERK
ERK
p-STAT3
STAT3

Fig.3E

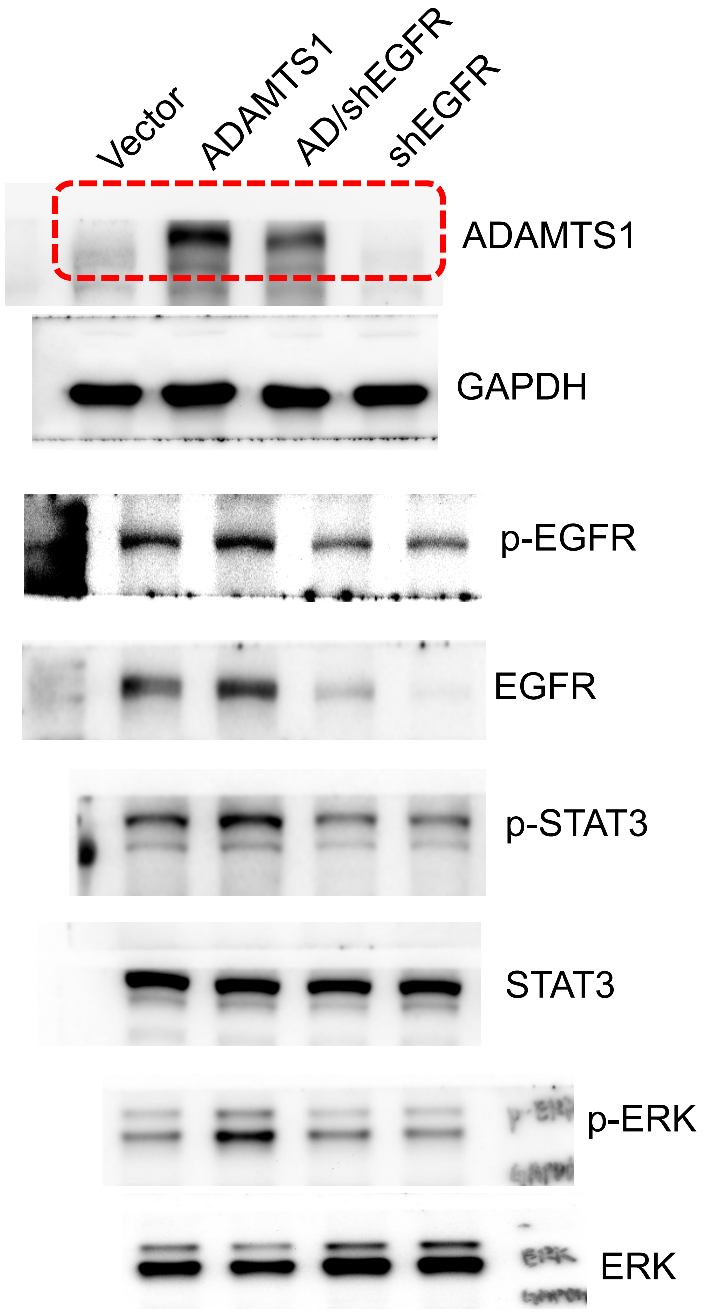
Vector
ADAMTS1
AD/shEGFR
shEGFR
ADAMTS1
GAPDH
p-EGFR
EGFR
p-STAT3
STAT3
p-ERK
ERK

Fig.4C

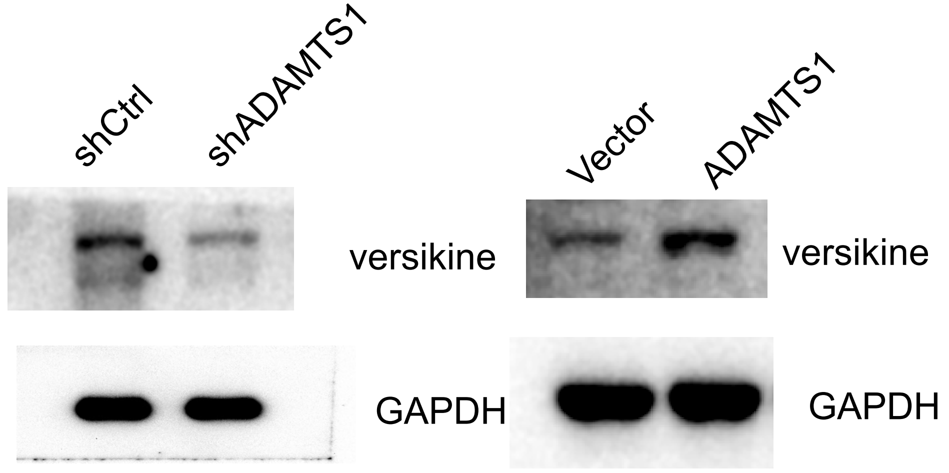
shCtrl
shADAMTS1
versikine
GAPDH
Vector
ADAMTS1
versikine
GAPDH

Fig.4F

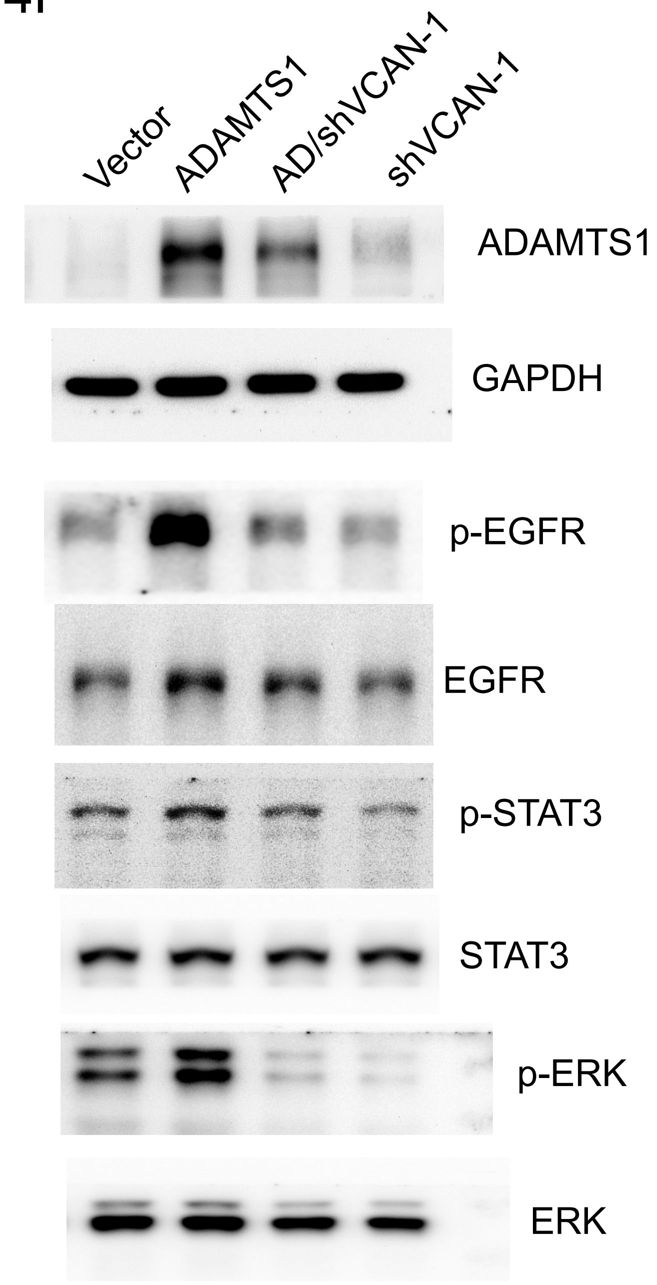
Vector
ADAMTS1
AD/shVCAN-1
shVCAN-1
ADAMTS1
GAPDH
p-EGFR
EGFR
p-STAT3
STAT3
p-ERK
ERK

Fig.5C

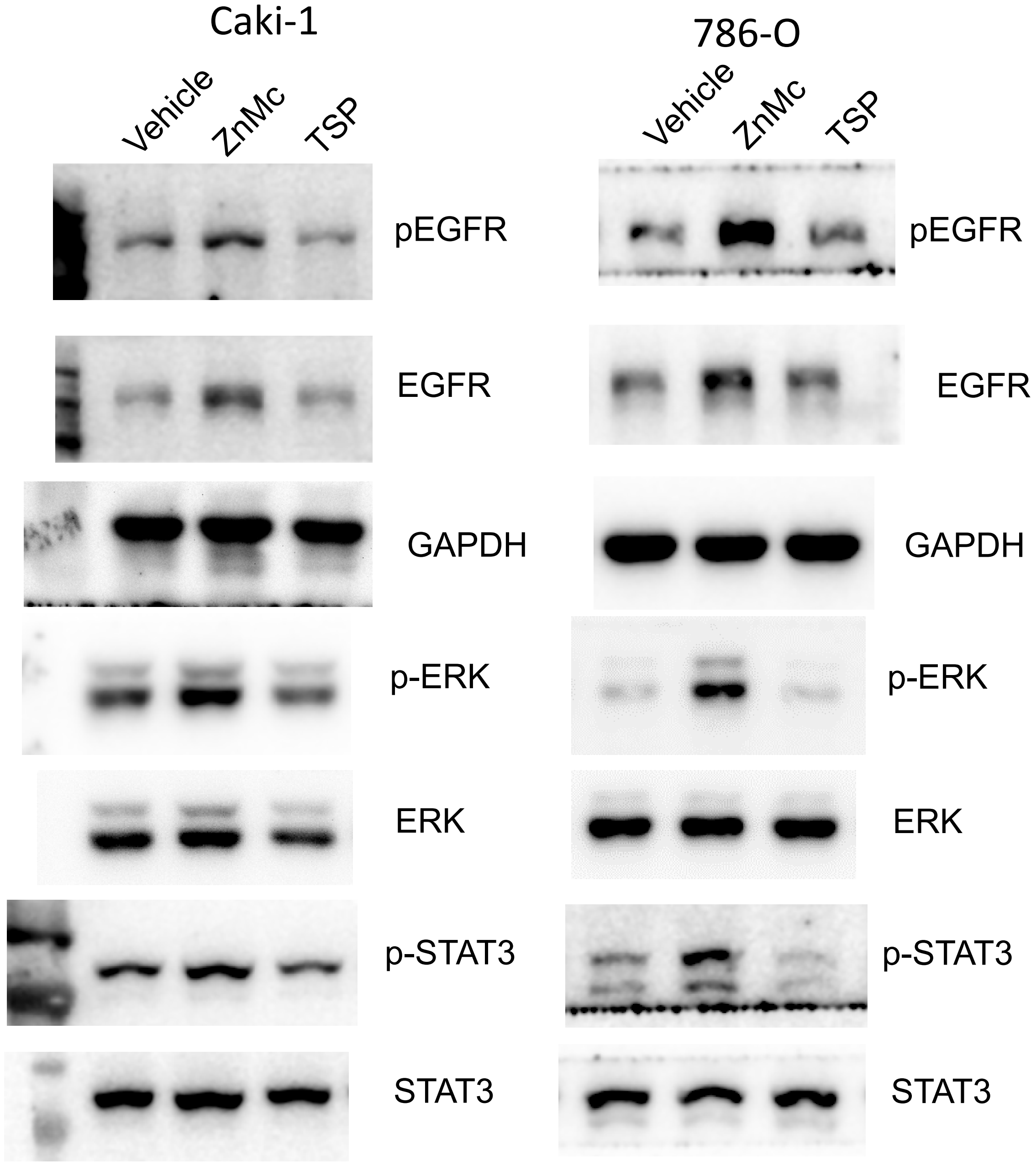
Caki-1
786-O
Vehicle
ZnMc
TSP
Vehicle
ZnMc
TSP
pEGFR
EGFR
GAPDH
p-ERK
ERK
p-STAT3
STAT3
pEGFR
EGFR
GAPDH
p-ERK
ERK
p-STAT3
STAT3

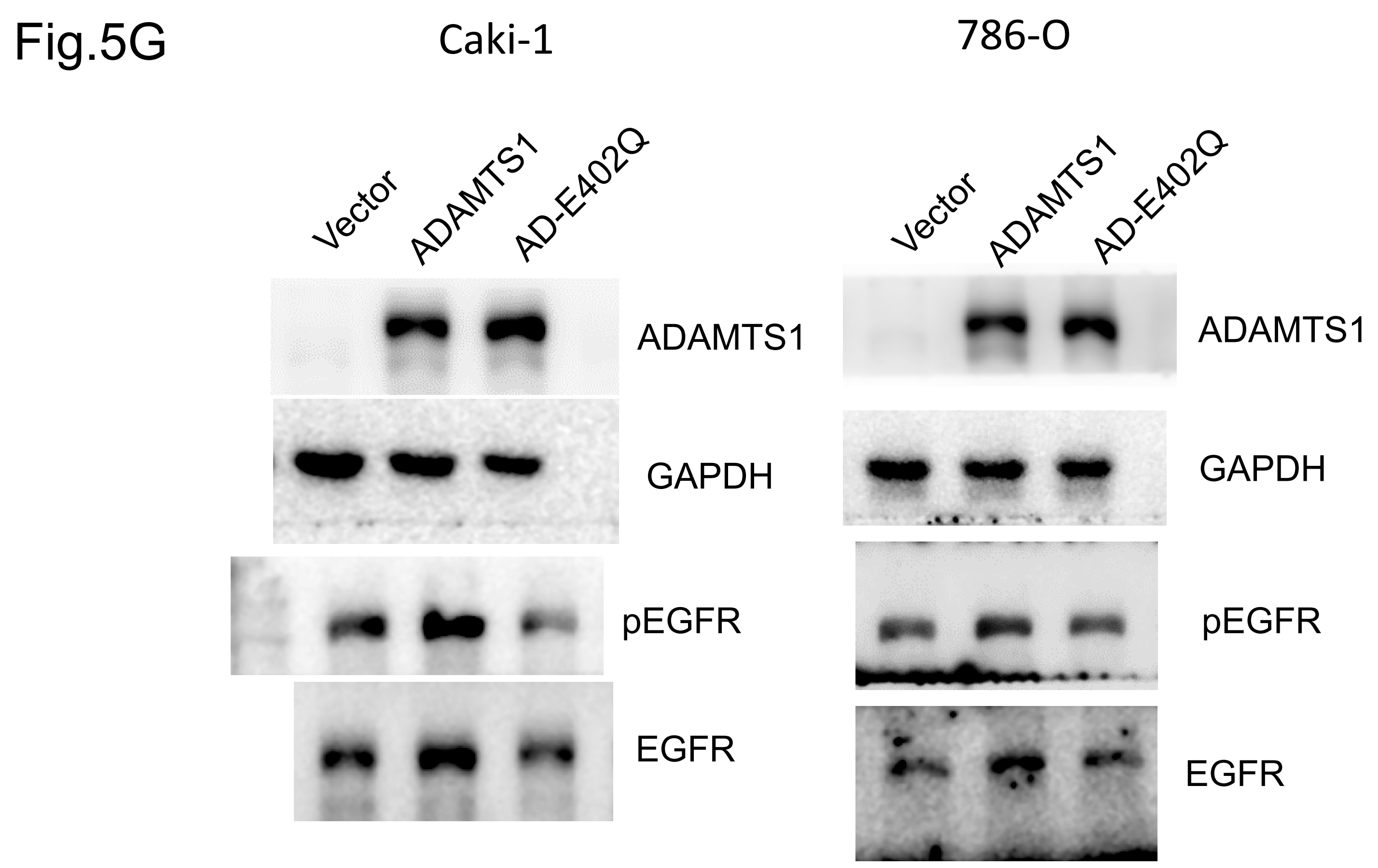
Fig.5G
Caki-1
786-O
Vector
ADAMTS1
AD-E402Q
ADAMTS1
GAPDH
pEGFR
EGFR

Fig.6C

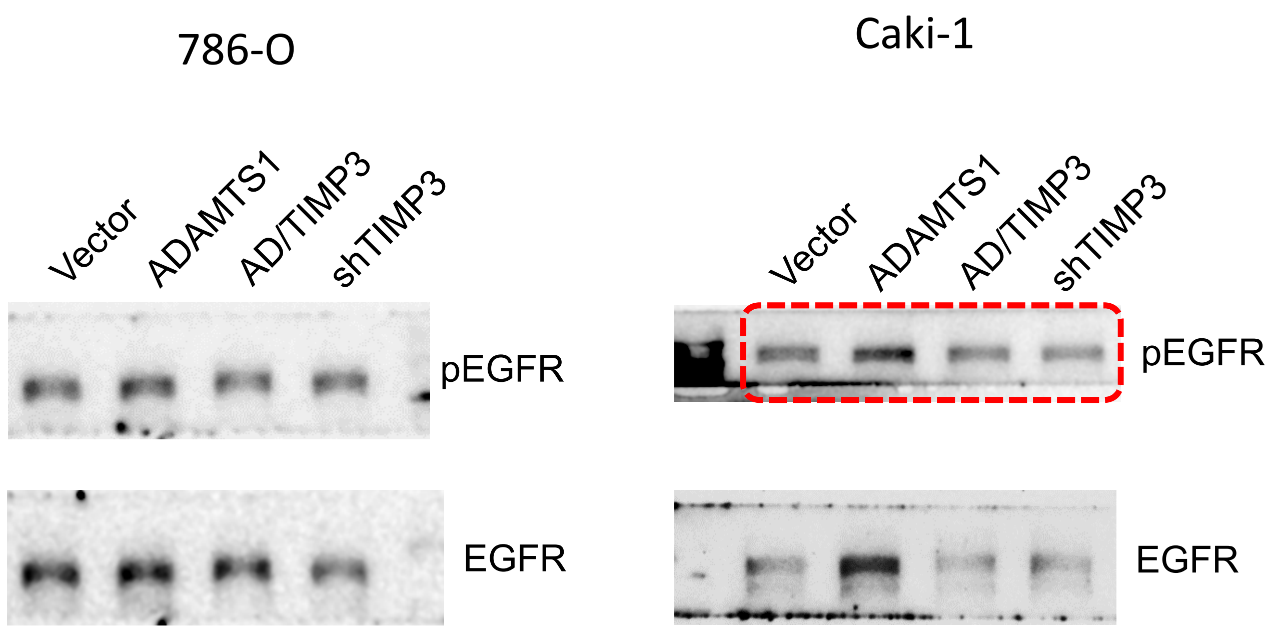

Fig.7C

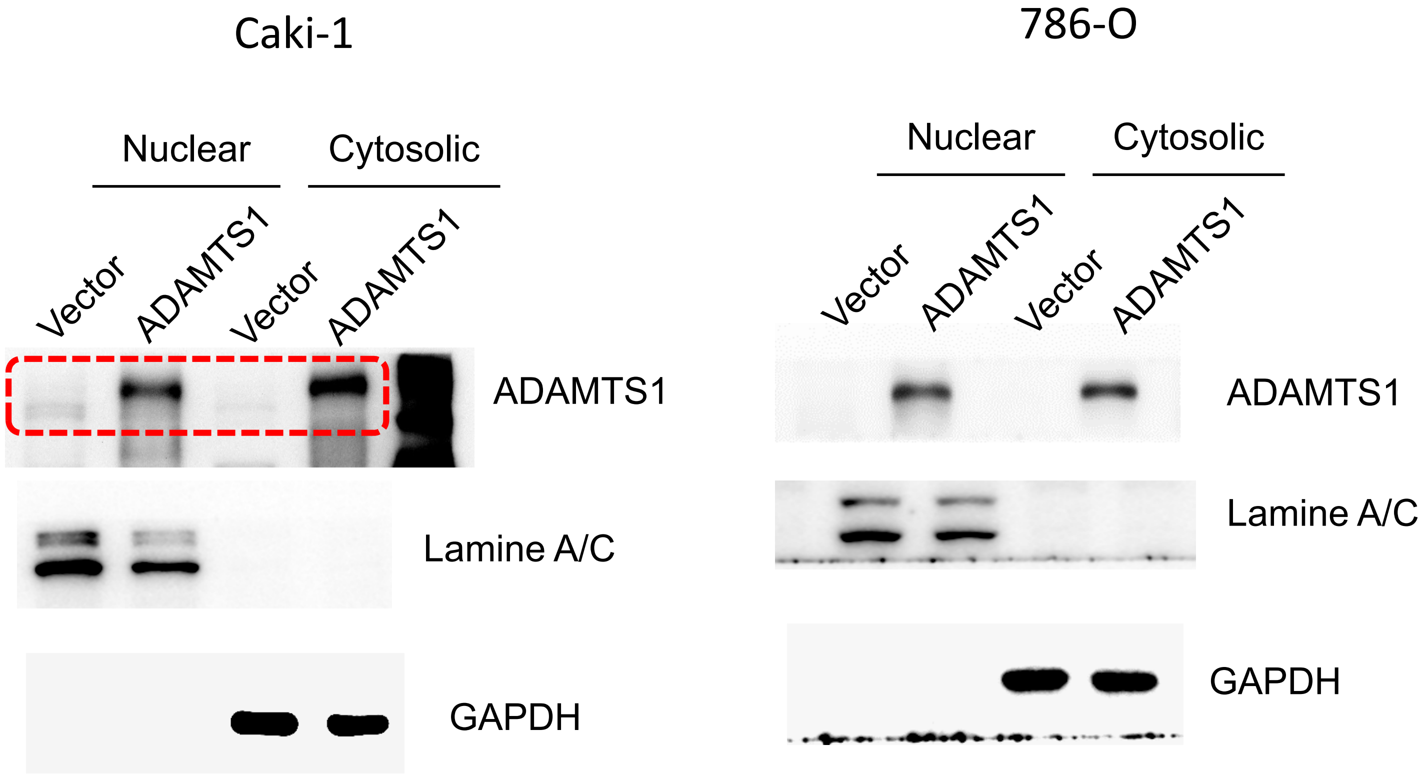

Caki-1
Nuclear
Cytosolic
Vector
ADAMTS1
Vector
ADAMTS1
ADAMTS1
Lamine A/C
GAPDH
786-O
Nuclear
Cytosolic
Vector
ADAMTS1
Vector
ADAMTS1
ADAMTS1
Lamine A/C
GAPDH

Fig.7D

Fig.7E

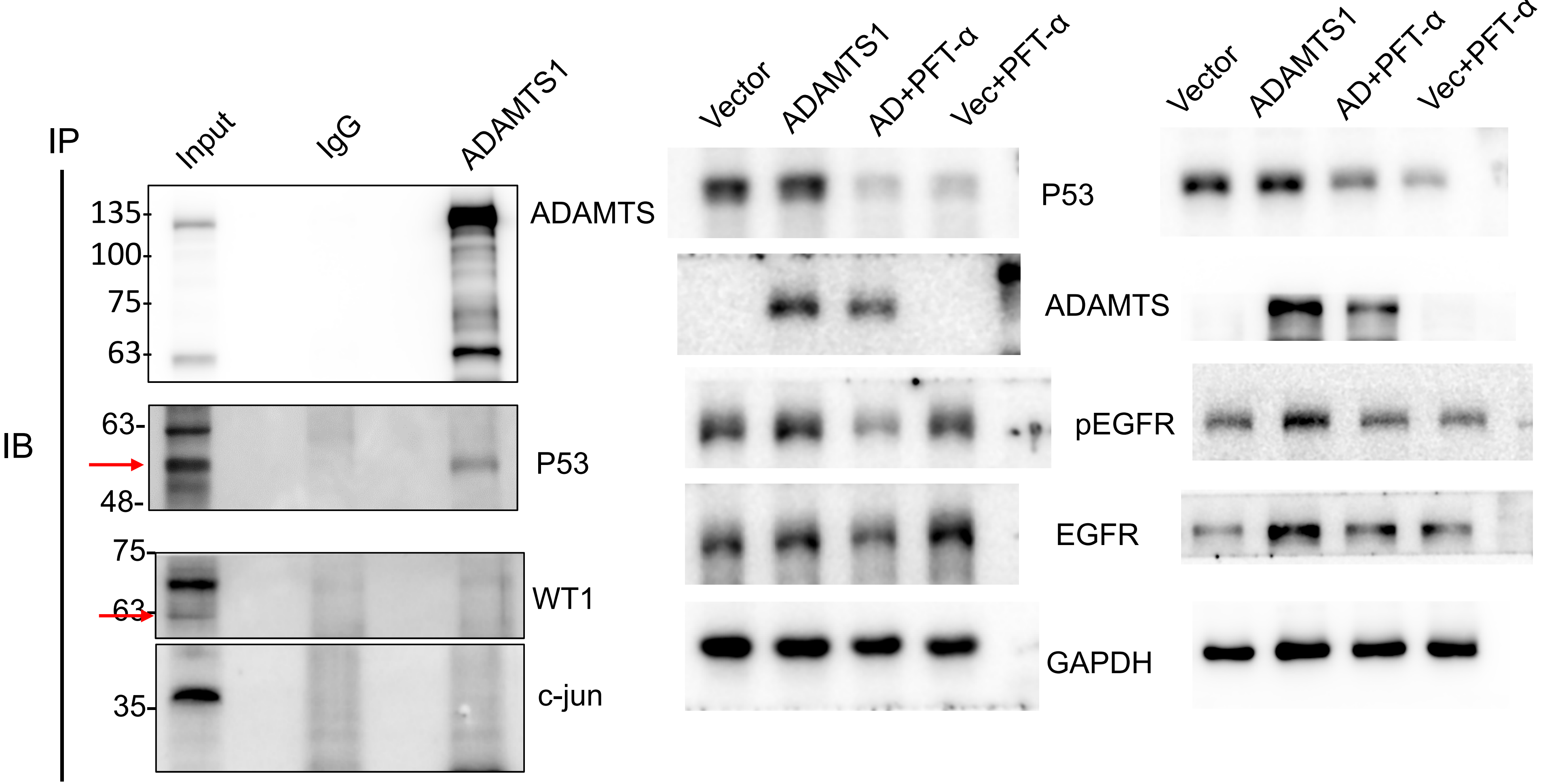
IP
Input
IgG
ADAMTS1
IB
135-
100-
75-
63-
ADAMTS
63-
48-
P53
75-
63-
WT1
35-
c-jun
Caki-1
786-O
Vector
ADAMTS1
AD+PFT-α
Vec+PFT-α
P53
ADAMTS
pEGFR
EGFR
GAPDH

Fig. S1

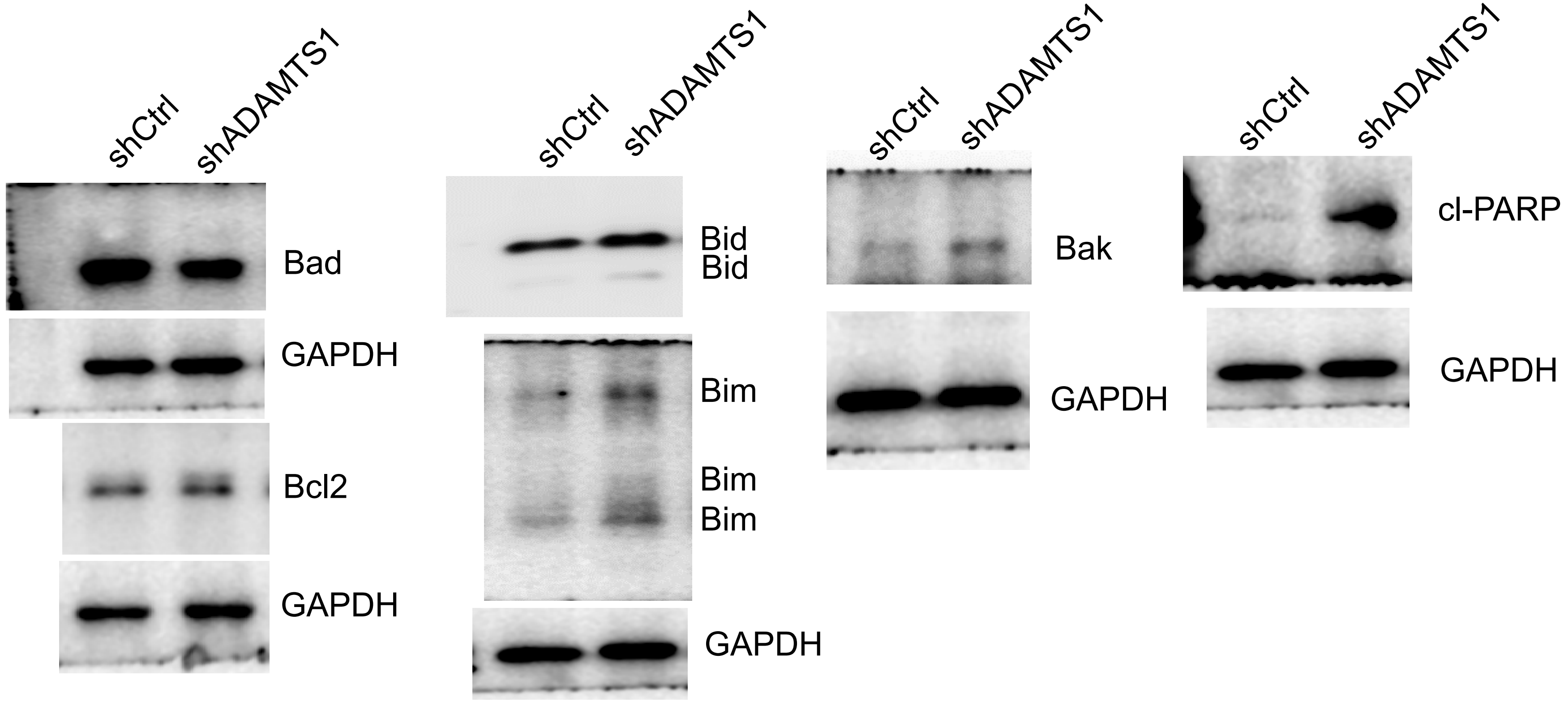

shCtrl
shADAMTS1
Bad
GAPDH
Bcl2
GAPDH
Bid
Bid
Bim
Bim
Bim
GAPDH
Bak
GAPDH
cl-PARP
GAPDH

Fig. S2A

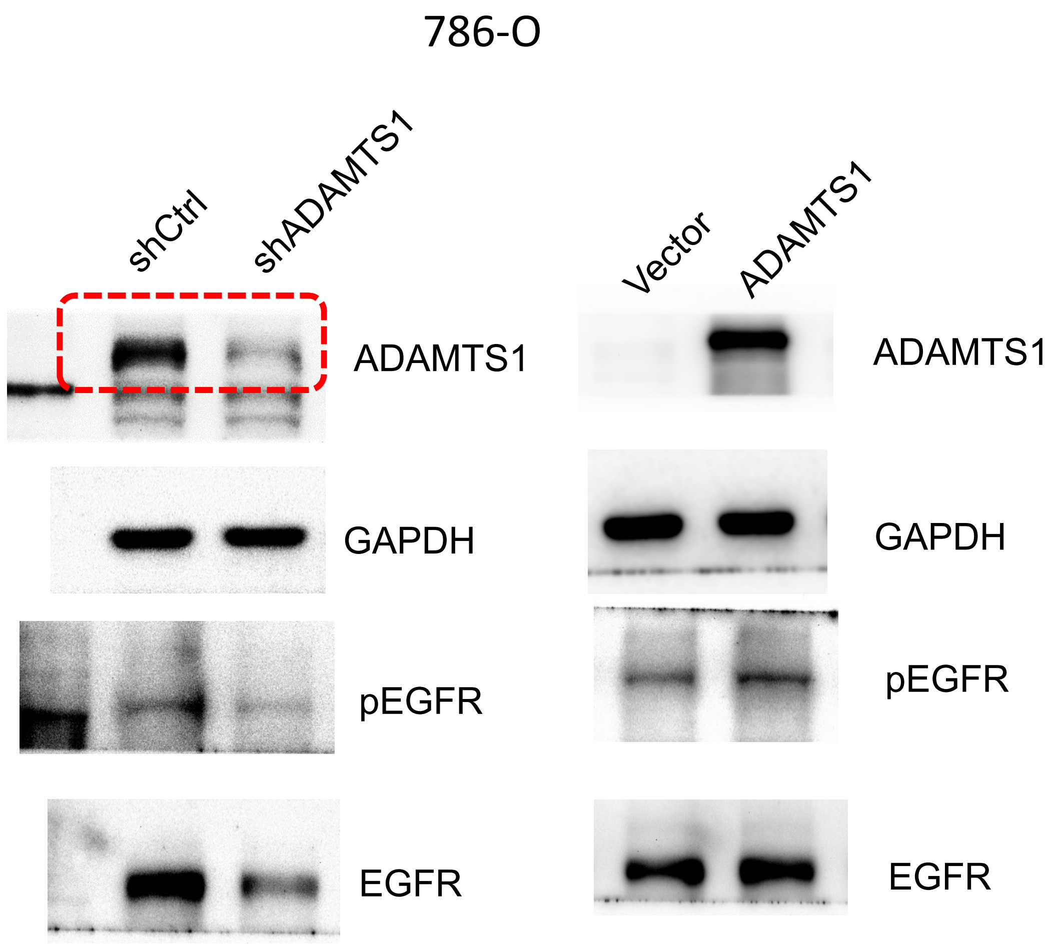

Fig. S2B

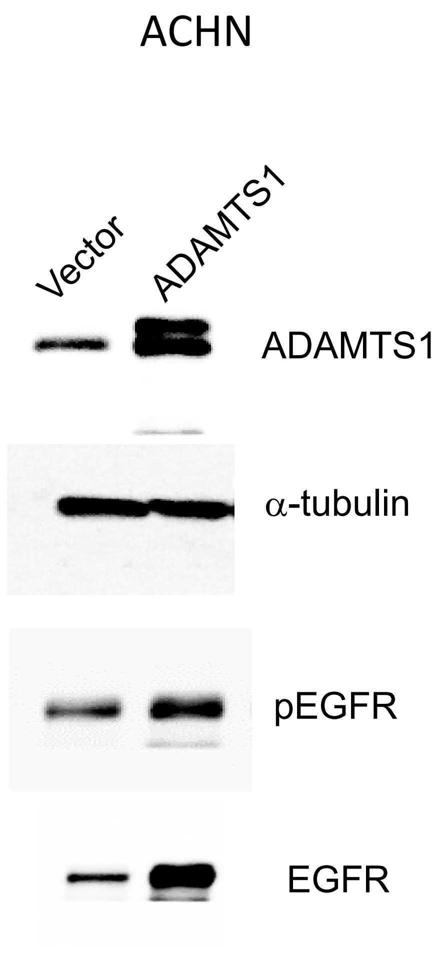

Fig. S2D

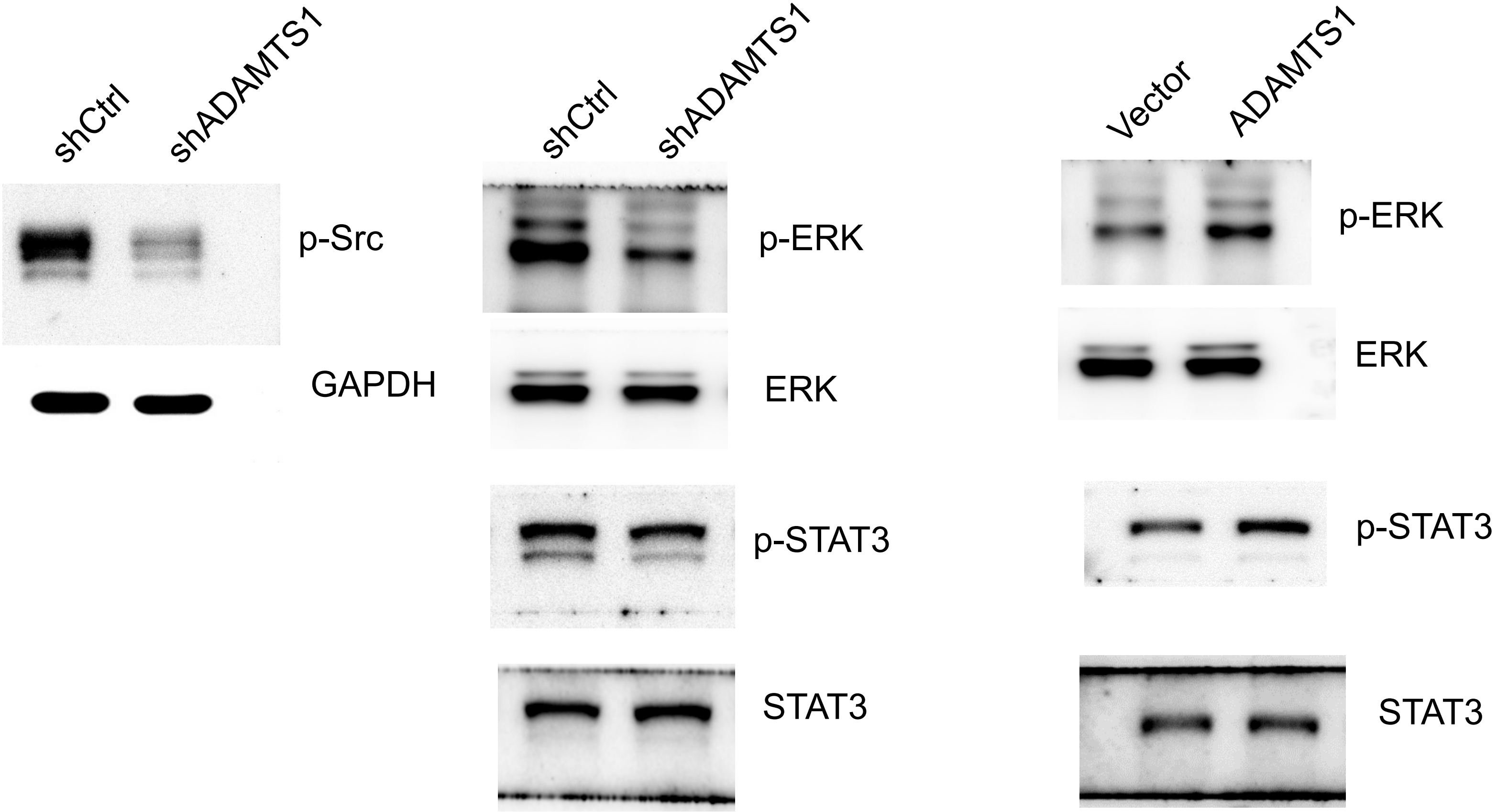
shCtrl
shADAMTS1
p-Src
GAPDH
shCtrl
shADAMTS1
p-ERK
ERK
p-STAT3
STAT3
Vector
ADAMTS1
p-ERK
ERK
p-STAT3
STAT3

Fig. S2E

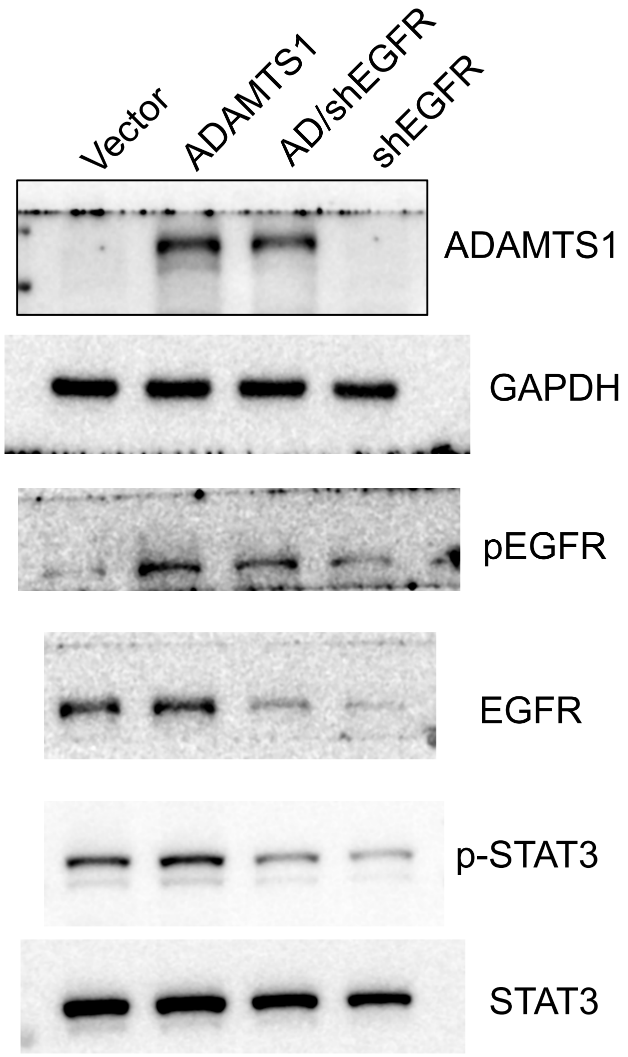

Fig. S4C

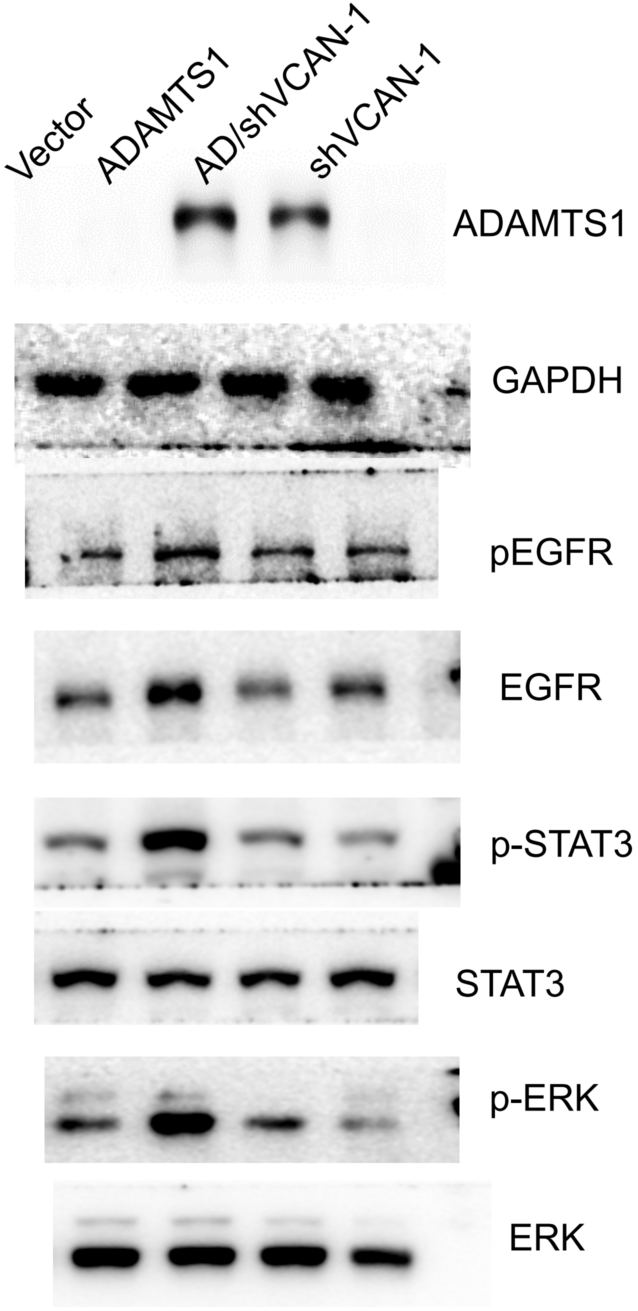

Fig. S4F

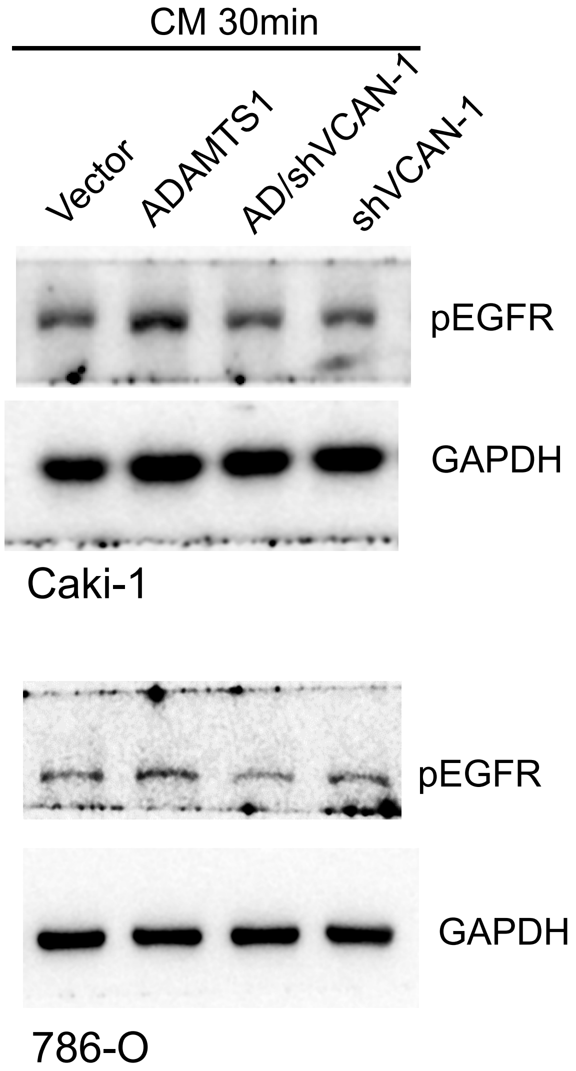

Fig. S6

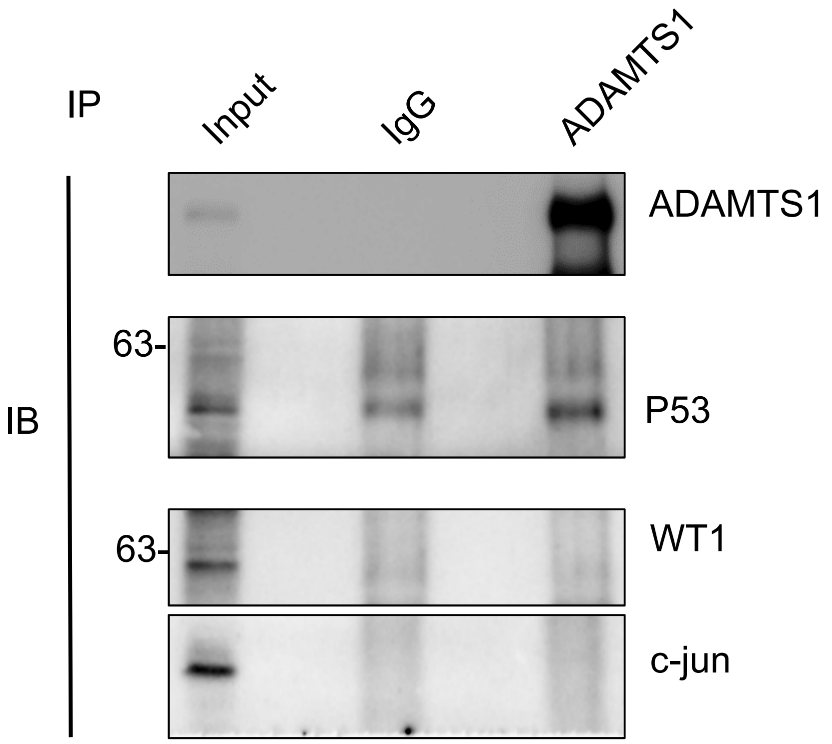

Fig. S7B

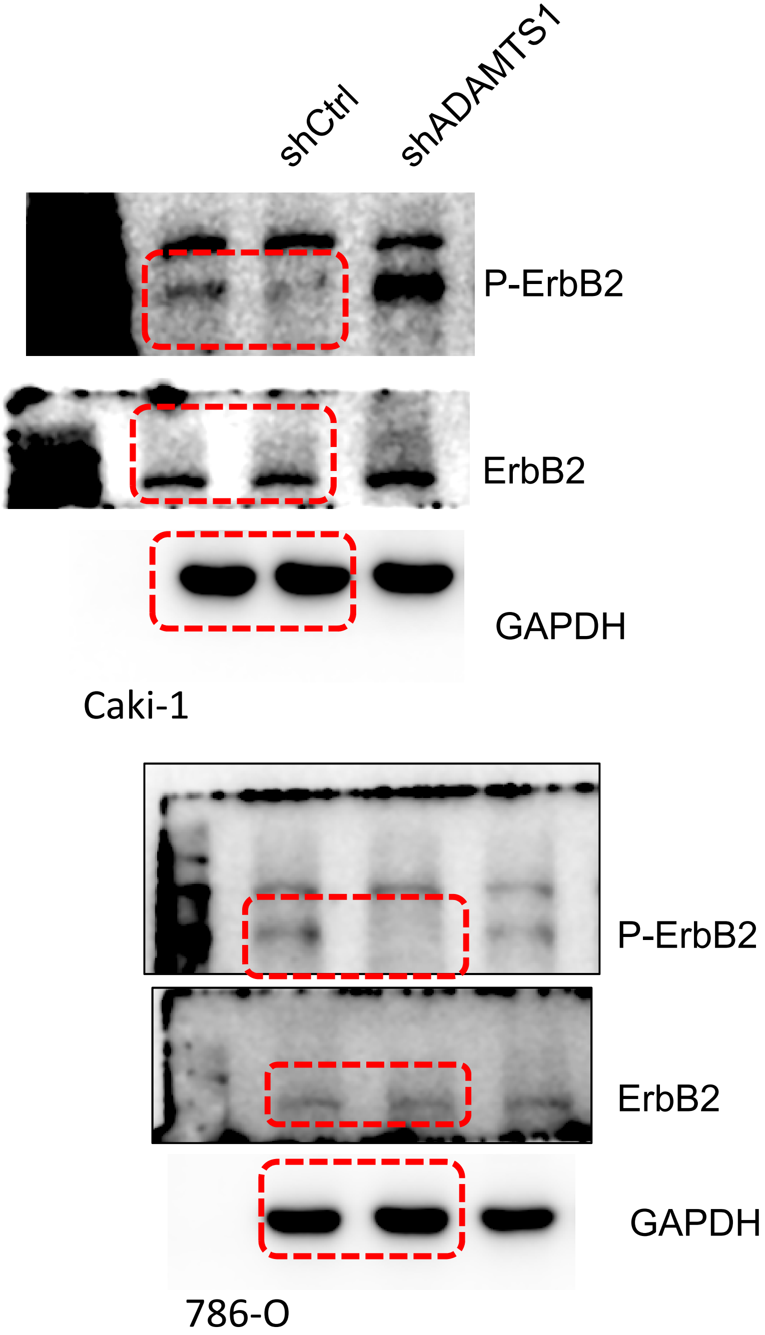

Supplement: Supplementary file 2 — Additional File 2. [file 11658_2024_643_MOESM2_ESM.pdf]
